# The Evolving E-cigarette: Comparative Chemical Analyses of E-cigarette Vapor and Cigarette Smoke

**DOI:** 10.3389/ftox.2020.586674

**Published:** 2020-12-15

**Authors:** Anthony Cunningham, Kevin McAdam, Jesse Thissen, Helena Digard

**Affiliations:** ^1^British American Tobacco (Investments) Limited, Research and Development, Southampton, United Kingdom; ^2^McAdam Scientific Ltd., Eastleigh, United Kingdom

**Keywords:** electronic cigarettes, nicotine salts, cotton wicks, HPHCs, NiFe coil, carbonyls, cigarette smoke toxicants

## Abstract

**Background:** E-cigarette designs, materials, and ingredients are continually evolving, with cotton wicks and diverse coil materials emerging as the popular components of atomisers. Another recent development is the use of nicotine salts in e-liquids to replicate the form of nicotine found in cigarette smoke, which may help cigarette smokers to transition to e-cigarettes. However, scientific understanding of the impact of such innovations on e-cigarette aerosol chemistry is limited.

**Methods:** To address these knowledge gaps, we have conducted a comparative study analyzing relevant toxicant emissions from five e-cigarettes varying in wick, atomiser coil, and benzoic acid content and two tobacco cigarettes, quantifying 97 aerosol constituents and 84 smoke compounds, respectively. Our focus was the potential for benzoic acid in e-liquids and cotton wicks to form aerosol toxicants through thermal degradation reactions, and the potential for nickel–iron alloy coils to catalyze degradation of aerosol formers. In addition, we analyzed e-cigarette emissions for 19 flavor compounds, thermal decomposition products, and e-liquid contaminants that the FDA has recently proposed adding to the established list of Harmful and Potentially Harmful Constituents (HPHCs) in tobacco products.

**Results:** Analyses for benzene and phenol showed no evidence of the thermal decomposition of benzoic acid in the e-cigarettes tested. Measurements of cotton decomposition products, such as carbonyls, hydrocarbons, aromatics, and PAHs, further indicated that cotton wicks can be used without thermal degradation in suitable e-cigarette designs. No evidence was found for enhanced thermal decomposition of propylene glycol or glycerol by the nickel–iron coil. Sixteen of the 19 FDA-proposed compounds were not detected in the e-cigarettes. Comparing toxicant emissions from e-cigarettes and tobacco cigarettes showed that levels of the nine WHO TobReg priority cigarette smoke toxicants were more than 99% lower in the aerosols from each of five e-cigarettes as compared with the commercial and reference cigarettes.

**Conclusions:** Despite continuing evolution in design, components and ingredients, e-cigarettes continue to offer significantly lower toxicant exposure alternatives to cigarette smoking.

## Introduction

Over the past 15 years, e-cigarettes have emerged into widespread use as credible alternatives to tobacco cigarettes. Vaping may offer a means of increasing adult cessation of combustible tobacco cigarettes, although there is also the risk of enhanced youth transition to combustible tobacco products (Stratton et al., 2018). In reviewing the scientific evidence base on e-cigarette safety, Public Health England have concluded that vaping carries lower risks than smoking (Public Health England, 2019). Consistent with this, studies of aerosol chemistry demonstrate substantial reductions in toxicant emissions in comparison to combustible tobacco cigarettes (Margham et al., 2016). In contrast, other reviews have concluded that the absolute risks of vaping cannot yet be determined unambiguously, noting evidence for DNA damage and mutagenesis from some aerosol components (Stratton et al., 2018), adverse events in the pulmonary, oral, gastrointestinal, and other bodily systems (Seiler-Ramadas et al., 2020), dependence arising from e-cigarette use, as well as hazards from battery explosions and incidence of fatalities associated with ingestion of e-liquids.

**Figure 1:**
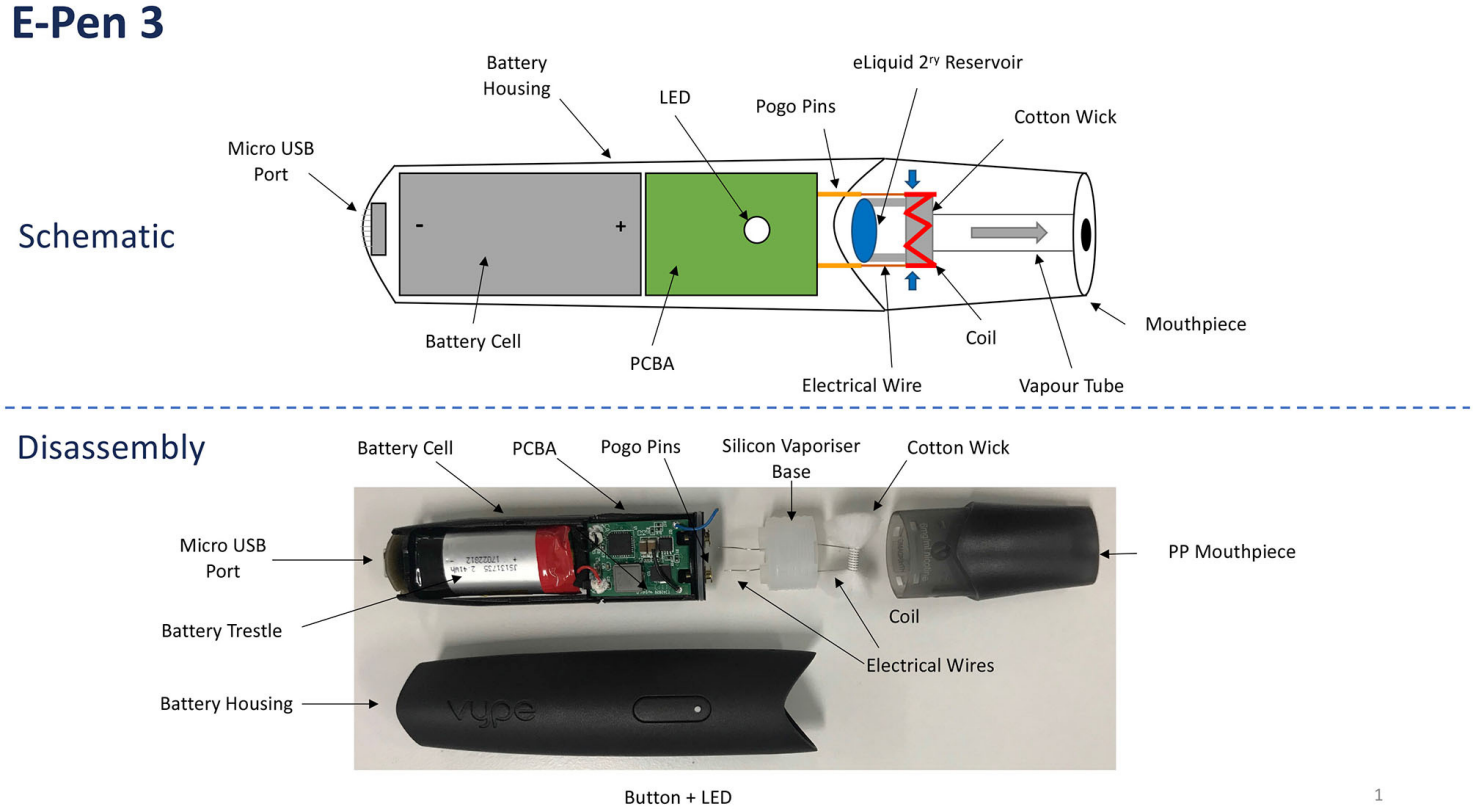
Schematic and image of the ePen3 device.

Given the relatively short time since their emergence, it is unsurprising that e-cigarettes continue to evolve in composition and performance (Malek et al., 2018). Despite the inevitable product diversity, however, all e-cigarettes share common attributes and performance traits. E-cigarettes comprise a reservoir of liquid (“e-liquid”), a transport system (“wick”) that carries the e-liquid from the reservoir to a heating (“coil”) zone (“atomiser”), a battery that supplies power to the coil, controlling electronics, and a mouthpiece, shown schematically in Figure 1. When activated, an e-cigarette functions by heating the e-liquid to its boiling point. The resulting gases are drawn away from the heated atomiser by the airflow created by the vaper's puff. The combination of rapid cooling, small particulate nucleation sites in the gas stream, and the presence of a supersaturated vapor causes the gases to condense into an aerosol cloud (“vapor”).

The e-liquid generally comprises glycerol (VG; boiling point [BP], 290°C) and/or propylene glycol (PG; BP, 188°C) as aerosol formers, plus a number of optional components including water as a viscosity controller; flavors for consumer appeal; and nicotine, the chief addictive agent in tobacco cigarettes and likely reason for how some smokers have switched from combustible cigarettes to e-cigarettes. Many studies have characterized the chemical composition of e-liquids and e-cigarette aerosols with considerable focus on their low-level toxicants. Several comprehensive integrated chemical studies have measured e-cigarette emissions of up to 142 analytes (Lauterbach and Laugesen, 2012; Lauterbach et al., 2012; Tayyarah and Long, 2014; Flora et al., 2016; Margham et al., 2016), identifying significantly lower levels of toxicants in e-cigarette aerosols than in cigarette smoke. By contrast, other studies have found much higher levels of toxicants, particularly VG and PG thermal decomposition products, in overheating and dry-wicking e-cigarette designs (Farsalinos and Gillman, 2018), demonstrating the need for careful thermal management in e-cigarettes.

A recent development in e-cigarette design has been the replacement of unprotonated nicotine in some e-liquids by nicotine salts. Nicotine is a di-basic compound (Clayton et al., 2013a,b) that reacts with acids in solution to form weak salts. Nicotine in tobacco and cigarette smoke is predominantly present in the mono-protonated form, complexed with multiple organic acids (John et al., 2018). Use of nicotine salts in e-cigarettes is proving popular with vapers, perhaps because the salts more faithfully mimic the chemical form of nicotine in cigarette smoke and are claimed to offer a “less harsh” experience during vaping (Strongin, 2019). Several organic acids have been tested for use in e-liquids (Bowen and Chenyue, 2015), but commonly used salts include nicotine benzoate and lactate. At e-cigarette operating temperatures, however, organic acids are often thermally unstable (Moldoveanu, 2010). In particular, polycarboxylic acids such as citric and tartaric acids thermally degrade to form toxic anhydrides. Benzoic acid (BA) is one of the more stable organic acids, but it also potentially decarboxylates at temperatures around 500°C, forming benzene or phenol (Moldoveanu, 2010). To our knowledge, only one study has examined toxicant formation from organic acids in an e-cigarette, reporting degradation of BA to benzene in a tank system used at possibly unrealistically high-power settings, however, benzene formation was not observed with a much lower powered cartomizer device (Pankow et al., 2017).

A further area of e-cigarette product evolution is the atomiser, which traditionally comprises a wick to transport the e-liquid from the reservoir to an electrically heated metal coil. The amount of e-liquid in the wick is critical in dictating the temperatures reached within the atomiser when the coil is heated (Chen et al., 2018). For example, temperatures of 145–334°C were recorded for an atomiser operating a conventional wicking process (typical of e-cigarette use) with a 100% PG test liquid (BP, 188°C); however, temperatures of 110–185°C were measured under extremes of wick loading (fully wet to fully dry) with an artificially fully wettened coil, while temperatures of 322–1,008°C were measured under artificial liquid-free conditions intended to replicate dry wicking.

The wick often comprises multiple strands of silica or cotton—two materials with significantly different properties. Cotton can transport e-liquid more efficiently to the coil, facilitating greater aerosol delivery to the vaper; however, it is less thermally stable than silica and may degrade if the coil temperature exceeds the decomposition threshold of cotton. In terms of chemical composition, cotton predominantly (>99%) comprises cellulose (Corradini et al., 2009; Liu, 2018), which when heated may liberate volatile organic compounds (e.g., aldehydes, acids, and esters) even at temperatures as low as 180°C during char formation steps (Yang and Freeman, 1993). As temperatures increase to 350°C and higher, aromatic compounds evolve from solid cellulosic char substrates, and benzene, toluene, naphthalene and anthracene are released from the char (Hajaligol et al., 2001). Polycyclic aromatic hydrocarbons (PAHs) are also pyrolysis products of cellulose at 300–650°C and may be formed via low-temperature mechanisms (McGrath et al., 2003). Above 600°C, carbon monoxide (CO) forms (Hajaligol et al., 2001). These observations are from cellulose-degradation experiments conducted under slow-heating conditions that are orders of magnitude slower than the temperature dynamics inside an e-cigarette atomiser. Reaction time and heating rate are critical parameters in thermal decomposition events; therefore, e-cigarette conditions are likely to be less favorable to thermal decomposition processes. Nevertheless, given the possibility of aromatic and PAH compound formation at the e-cigarette operating temperature range, it is important to test whether they are formed with commercial e-cigarettes.

Lastly, the atomiser coil, which commonly comprised an alloy such as nichrome (NiCr) in early e-cigarettes, now can comprise of kanthal, nickel–iron (NiFe), stainless steel or pure metals such as nickel or titanium. Notably, metal catalysis has been suggested to enhance the thermal decomposition of PG and VG (Jensen et al., 2017), PG and VG may interact with various metal surfaces (Tuma et al., 2013), and the coil material has been shown to affect PG decomposition in a heated flow reactor (Saliba et al., 2018). These observations suggest that some coil materials may interact with the e-liquid, degrading the aerosol formers in the atomiser. Despite this possibility, it is currently unclear whether metal coil materials influence toxicant production from e-liquids to any significant extent under real-world usage conditions.

Paralleling the changes in e-cigarette design, regulatory lists of toxicants are also evolving in response to evidence of toxicants in e-cigarette aerosols. As part of e-cigarette pre-launch product registration and reporting requirements in Europe, the Tobacco Product Directive now stipulates chemical emissions testing for multiple priority compounds, including acetaldehyde, acrolein, and formaldehyde (EU, 2014). Dependent on several factors, reporting of diethylene glycol, ethylene glycol, diacetyl, pentane-2,3-dione, and tobacco-specific nitrosamines (TSNAs) emissions may also be required. Metals including aluminum, chromium, iron, nickel, and tin are also stipulated for reporting, as well as lead and mercury if present in the e-cigarette device (UK Emissions Testing Guidance, 2016).

The US FDA has established a list of more than 90 Harmful and Potentially Harmful Compounds (HPHCs) in tobacco products (FDA, 2012), and recently sought public comment on the proposal to add a further 19 compounds to the list (FDA, 2019). Among these compounds, glycidol, a probable human carcinogen (IARC, 2000), which is a thermal decomposition product of VG (Laino et al., 2011; Sleiman et al., 2016). Ethylene glycol has been found in e-liquids (Hutzler et al., 2014) and has adverse respiratory effects on inhalation. Diethylene glycol, when identified in e-liquids and aerosols, is thought to arise as a contaminant of the VG or PG stocks used by e-liquid manufacturers (Varlet et al., 2015); it may induce severe and irreversible acute toxic affects (Sanina, 1968; Health Council of the Netherlands, 2007; Australian Government Department of Health and Ageing, 2009; Schep et al., 2009; California Poison Control System, 2012; Devoti et al., 2015), and has been identified by the Californian EPA as a reproductive toxicant if ingested (Borghardt et al., 2018). According to the National Institute for Occupational Safety and Health (NIOSH), the remaining 16 toxicants (acetic acid, acetoin, acetyl propionyl, benzyl acetate, butyraldehyde, diacetyl, ethyl acetate, ethyl acetoacetate, furfural, VG, isoamyl acetate, isobutyl acetate, methyl acetate, *n*-butanol, propionic acid, and PG) have adverse respiratory effects. At present, however, there are few data on the emissions of these 19 additional HPHCs, and validated analytical methods for their quantification are not widely available.

It is of considerable interest to compare e-cigarette toxicant emissions with those of combustible cigarettes. Previous comparisons of this kind have largely focused on per-puff measurements, due to the differences in usage patterns between cigarettes and e-cigarettes. As estimates for puffs per day from e-cigarettes (e.g., mean 163 ± 138, median 132 Dautzenberg and Bricard, 2015), and combustible cigarettes (estimates of average values of 14 cigarettes per day with puffs/cigarette around 10) are broadly comparable this approach appears reasonable. However, additional factors may be important to consider, such as compensatory behavior amongst vapers. For example, Dawkins et al. (2018) examined the effects of differing e-liquid nicotine concentrations and device power levels on e-cigarette consumption. They identified evidence for compensatory behaviors amongst vapers where use of a lower nicotine concentration e-liquid may be associated with higher number and duration of puffs as well as formaldehyde exposure. Similarly, Farsalinos et al. (2018) identified compensatory puffing patterns and nicotine self-titration, resulting in a change in puffing patterns (puff number and duration) when vapers change the power settings of an e-cigarette device. These observations suggest that it is also important to consider differences in toxicant emissions as a function of nicotine delivery when comparing emission data from e-cigarettes and combustible cigarettes of differing nicotine content.

The purpose of the present study was to understand whether recent developments in e-cigarette product design influence aerosol emissions, particularly those that may arise from use of two thermally sensitive materials: cotton and BA, and a relatively new coil material, NiFe. In addition, two modern e-cigarette designs have been quantitatively characterized for emissions of the FDA's proposed 19 additional HPHCs, in order to understand this poorly understood area of aerosol chemistry. We contextualize the emissions against values for smoke yields from two cigarettes, a commercial cigarette and a reference product, as well as background air/method baseline values from the measurement laboratory. We also examined the impact of comparing emissions data per-puff and per-mg of nicotine to understand the potential impact of compensatory puffing behavior that might occur when vapers use differing nicotine content e-cigarettes.

## Methods

### Test Products

#### Cigarette Comparators

For comparison, two cigarette products were used: Kentucky reference 1R6F (Jaccard et al., 2019), a king-size cigarette with US-style blended tobacco (ISO tar yield, 9.3 mg) and a cellulose acetate filter; and Benson & Hedges Skyblue (Japan Tobacco International), a king-size commercial cigarette with US-style blended tobacco (length, 83 mm; circumference, 24.2 mm; weight, 0.82 g; ISO tar yield, 8.7 mg) and a 21 mm cellulose acetate filter with 30 mm filter tipping and 36% filter ventilation. The cigarette paper of Benson & Hedges Skyblue (B&H Skyblue) is banded: the air permeability is 87 mL/min/cm^2^ between bands and 6.72 mL/min/cm^2^ on the bands.

#### E-Cigarette Devices

Two e-cigarette devices were tested: Vype ePen2 (Nicoventures Trading Ltd., Blackburn, UK) and Vype ePen3 (Nicoventures Trading Ltd.). Vype ePen2 consists of a reusable section containing a 650-mAh rechargeable battery and an actuation button, a disposable flavor cartridge and a mouthpiece cover. It uses a silica rope wick, and an NiCr coil. The device comes with two power settings (high, 4.4 W; and low, 2.8 W); the high-power setting was used in this study.

Vype ePen3 has a different design to the ePen product used in an earlier study (Margham et al., 2016) and is shown schematically in Figure 1. It comprises a “closed system” e-cigarette with a rechargeable battery and a flavored e-liquid pod of 2-mL capacity. The device measures 121 × 26 × 12 mm and weighs 39 grams with a full pod. The e-cigarette is powered by a 650-mAh battery, which is connected to a coil with resistance of 1.95–2.36 ohm, resulting in a power output of 5.9 W. The battery electronics has a protect circuit board (PCB) to protect against short-circuiting, low or high charging voltage, over current, and over charging. The PCB stops battery power to the coil after 8 s, thereby limiting dry-puff events, and causes the device to automatically power off after 10 min of inactivity. The coil is made from a NiFe alloy whose resistance is strongly temperature dependent. The 5.9 W power rating of the device was delivered at the operating temperature and resistance, with device electronics monitoring power as a function of voltage. The device uses a cotton wick to transport e-liquid to the heated coil.

The device was tested for electrical safety performance and was compliant with the essential requirements of the following applicable CE marking European Directives: 2014/30/EU Electromagnetic Compatibility (EMC) Directive 2011/65/EU; and Annex II Amendment (EU) 2015/863 Restriction of the Use of Certain Hazardous Substances in Electrical and Electronic Equipment (RoHS). Conformity was assessed in accordance with the following harmonized EMC standards: Requirements for Household Appliances, Electric Tools and Similar Apparatus, Part 1 Emission (EN55014-1 and CISPR 14-1) and Part 2 Immunity (EN55014-2 and CISPR 14-2); and Product Family Standard for Aftermarket Electronic Equipment in Vehicles (EN 50498). Conformity was also assessed in accordance with the following harmonized RoHS standards: Technical Documentation (EN 50581, IEC 63000); and Determination of Certain Substances (IEC/EN 62321-1). In addition, the device was certified for low-voltage electrical safety within the IECEE CB Scheme: Household and Similar Electrical Appliances—Safety, Part 1 General Requirements (IEC 60355-1).

A fully charged ePen3 battery provides ~200 puffs (based on an 80-mL, 3-s puff taken once every 30 s), which matches the liquid capacity of the pod under these testing conditions.

#### E-Liquids

Five e-liquids with two variants of tobacco-style flavor (“Blended Tobacco” and “Master Blend”) of different compositions were used in this study (Table 1). PG, VG, nicotine, and water were of pharmacopeia standard purity. The flavor compounds were a minimum of food grade and their safety in an inhalation context was evaluated by following Product Stewardship principles (Costigan and Meredith, 2015), and (Costigan and Lopez-Belmonte, 2017). In all cases, the compound flavors accounted for up to 1% of the e-liquid formulation.

**Table 1:** E-liquid composition of the study products.

**E-cigarette description**	PG % (w/w)^*^	**VG % (w/w)**	**Water % (w/w)**	**Nic % (w/w)**	**BA level**	**Flavor type**
ePen2 18 mg/mL Nic BT	25.00	48.22	25	1.78	0	Blended Tobacco
ePen3 18 mg/mL Nic BT	54.00	34.22	10	1.78	0	Blended Tobacco
ePen3 12 mg/mL Nic Low BA	54.25	34.57	10	1.18	Low	MasterBlend
ePen3 18 mg/mL Nic Medium BA	54.73	33.5	10	1.77	Medium	MasterBlend
ePen3 30 mg/mL Nic High BA	56.06	31.2	10	2.74	High	MasterBlend

*BA, benzoic acid; Nic, nicotine*.

* *Reported propylene glycol content also includes % content of flavor compounds and benzoic acid*.

Blended Tobacco (BT) was tested in ePen2 and ePen3, which differ in coil and wick type, and operating power. Due to the differences in e-cigarette wicks between ePen2 and ePen3, the same PG/VG ratio cannot be used in both products. This is because the silica wick of ePen2 had inferior wicking properties toward high viscosity liquids compared to those of the cotton wick in ePen3. Hence the e-liquid water content was higher in the ePen2 e-liquid than in ePen3, to reduce the liquid viscosity to functional levels. Comparison of the aerosol chemistry between these two products therefore reflects the potential effects of three factors: (1) silica vs cotton wicks; (2) differences in PG/VG/water ratios; and (3) differences in coil power, where ePen3 > ePen2.

Master Blend (MB) was tested in three ePen3 e-liquids differing only in nicotine and BA content (which was increased by substitution with VG in the formulation): 12 mg/mL nicotine with low levels of BA; 18 mg/mL nicotine with medium levels of BA; and 30 mg/mL nicotine with high levels of BA. Comparison of the aerosol chemistry among these three products therefore reflects the combined influence of increasing nicotine/BA content and ratio, and small changes (~10%) in VG content (from 32.1 to 34.5% in the formulation).

Comparison of the aerosol chemistry between ePen3 Blended Tobacco (18 mg/mL nicotine) and ePen3 Master Blend (18 mg/mL nicotine with medium BA) also provides insight into the influence of BA, together with the effect of a small change in VG level and flavor type.

### Puffing Conditions

Prior to testing, the commercial and reference cigarettes were marked with the standard butt length as specified in ISO 4387 (2000). Cigarettes were conditioned and tested under a conditioned laboratory environment of 22 ± 2°C and 60 ± 5% relative humidity as specified in ISO 3402 (1999). Tobacco cigarettes were smoked on a rotary or a linear smoking machine using “Canadian Modified” conditions (55-mL puff volume, 2-s puff duration, 30-s interval, vents blocked) (ISO 20778, 2018).

E-cigarette samples were puffed in a dedicated e-cigarette room under a conditioned laboratory environment of 22 ± 2°C and 60 ± 5% relative humidity as specified in ISO 3402 (1999). Puffing of e-cigarettes was carried out on a linear smoking machine using an automated e-cigarette activation system and puffing parameters set out in the CORESTA Reference puffing method CRM81 (CORESTA, 2015) and ISO 20768 (2018) (55-mL puff volume, 3-s puff duration, 30-s interval, square wave puff profile, no ventilation blocking).

### Emissions Analysis

Cigarette smoke toxicants and e-cigarette emissions were measured by using Labstat standard methods, as described previously (Margham et al., 2016). The 19 additional HPHCs proposed by the FDA were measured in e-cigarette aerosol using the following methods. Aromatic flavourants were determined in emissions from e-cigarettes by using Labstat method TMS-00175. In brief, e-cigarette aerosol was generated by an automated constant volume linear smoking machine and target compounds were trapped on a 44-mm glass fiber filter disc (pad) followed by a cryogenic (≤-35°C) trap (impinger) containing 20 mL of acetonitrile. The pad was folded, placed in a 25-mL amber glass vial, combined with the impinger solution and extracted for 30 min by using a platform shaker. A 5-mL aliquot of the extract was added to 50 μL of internal standard (ISTD) solution. The sample was then analyzed by gas chromatography–mass spectrometry (GC-MS).

Propionic acid was determined in e-cigarette aerosol by using Labstat method TMS-00177. In brief, e-cigarette aerosol was generated and emissions were trapped on a pad and impinger as described for TMS-00175. The pad was combined with the impinger solution and an internal standard solution (Anisole) and extracted by using a platform shaker. The extract was analyzed by selective ion monitoring (SIM) GC-MS using a WAX-type capillary column.

Acetic acid was determined in e-cigarette aerosol by using Labstat method TMS-00115A. In brief, e-cigarette aerosol was generated by using a linear smoking machine and emissions were collected on a 44-mm Cambridge filter pad. The pad was extracted with 20 mL of 0.1% H_3_PO_4_ by shaking for 45 min. The extract was then analyzed by HPLC-UV using a C_18_ column with detection at 210 nm. Owing to a lack of established methods applicable to smoke analysis, not all of the additional HPHCs were evaluated for the comparator cigarettes.

For all analyses, 50 puffs of ePen3 or ePen2 were used per collection, which is half the puff number used in previous studies (Margham et al., 2016), because ePen3 delivers approximately twice as much aerosol mass per puff. Air/method blank determinations were also conducted in order to identify background contaminants and analytical artifacts. In all cases 5 replicates were measured per observation.

### Accelerated Aging Tests for Metal Emissions

Metal emission measurements were conducted to examine the potential for benzoic acid in the e-liquids to corrode the metallic elements of the e-cigarettes, and increase aerosol metal emissions. Metal corrosion by acids is a time-sensitive phenomenon, and therefore we conducted accelerated aging tests where sealed cartomisers containing unflavoured e-liquids and various levels of benzoic acid and nicotine were stored at 40°C/75% RH for 3 months prior to testing. The accelerated conditions were selected to offer a means of reproducing typical shelf-life times for e-cigarettes. After aging the cartomisers were allowed to stabilize at laboratory testing conditions prior to measurements being made. Comparator cigarettes were not subject to accelerated aging conditions prior to testing.

### Data Treatment and Analysis

For measurable analytes, data were reported as mean ± SD. To facilitate comparisons between e-cigarette aerosol and cigarette smoke, the data were treated as follows. We compared data both on a per-puff basis, where measurements per collection were divided by the puff number, and per-nicotine where we divided the toxicant emission values by the average nicotine emission value. Where values were less than the limit of detection (<LOD) or limit of quantification (<LOQ), we imputed a value of LOD/2 or the midpoint between LOD and LOQ, respectively, as described previously (Margham et al., 2016). Comparisons were made based on mean per-puff data using the derived values for <LOD and <LOQ values where necessary. Comparisons were only made where the overall per collection mean (based on derived values) for a given analyte was above the LOQ for at least one product.

We calculated the percent reduction in emissions from all five e-cigarettes relative to smoke from both cigarettes except where both e-cigarette and cigarette mean values were <LOQ. Percent reductions above 99.9% were reported as >99.9%.

Because the results of the percent reductions were sensitive to the imputed values used in the calculations, we assessed the magnitude of errors that might arise from use of the midpoint approach, by comparing the percent reductions estimated by this method with those obtained by using two “boundary condition” approaches.

An “upper boundary” estimate approach, where <LOQ values are imputed as the LOQ, and <LOD values are imputed as the LOD, reflecting the maximum possible concentrations of an unquantifiable compound that may be present in the analyzed sample.

A “lower boundary” estimate approach, where values <LOD are imputed as zero, and values <LOQ are imputed as the LOD, reflecting the minimum possible concentrations of an unquantifiable compound that may be present in the analyzed sample.

The impact of the three imputation strategies on the calculated percent reductions was assessed for the nine cigarette smoke analytes prioritized for reduction by the World Health Organizations Tobacco Product Regulation advisory group (WHO TobReg) (Burns et al., 2008).

Statistical comparisons are made between test product and blank emission yields on a per puff basis. When comparing between test products, Generalized Linear Models (GLMs) are used with *post-hoc* Tukey adjustment with an alpha of 0.05. Alternatively, when comparing multiple test products to the air blank yield, GLMs are used with Dunnett's control adjustment with an alpha of 0.05. When two yields are evaluated (test product or air blank), independent samples *t*-tests are used for the statistical comparison.

Statistical comparisons are also made on a per milligram of nicotine basis. This was done by taking the average nicotine per puff value of each of the products and divide the constituent measurement per puff by the average mg of nicotine per puff. Comparisons are made between the reference products and test products. These comparisons do not include blanks, as nicotine was not measured in the blanks. When comparing the test products and reference products, GLMs are used with *post-hoc* Tukey adjustment with an alpha of 0.05.

## Results

Overall, the aerosols from five e-cigarette variants (Table 2) and mainstream smoke from two conventional cigarettes (Table 3) were analyzed for 97 and 84 potential toxicants, respectively, together with air/method blanks as a control. The data in Tables 2, 3 are presented on a per-puff basis, however we also present the data for those quantified analytes on a per-nicotine basis in Table 4. Table 5 presents the results of the accelerated aging study of metals emissions. Below, we describe the findings for each group of analytes.

**Table 2:** Per-puff emissions of components from the e-cigarettes and air/method blank.

**Aerosol constituent**	**Unit**	**LOD**	**LOQ**	**Air/method blank**		**ePen2 18 BT**		**ePen3 18 BT**		**ePen3 MB 12 Low BA**		**ePen3 MB 18 Medium BA**		**ePen3 MB 30 High BA**	
				**Mean**	**SD**	**Mean**	**SD**	**Mean**	**SD**	**Mean**	**SD**	**Mean**	**SD**	**Mean**	**SD**
Carbon monoxide	μg/puff	10.50	34.99	BDL	BDL	BDL	BDL	BDL	BDL	BDL	BDL	BDL	BDL	BDL	BDL
**ACM, water, and nicotine**															
Water	μg/puff	3.83	12.75	BDL	BDL	1,014	144.0	1,090	36.00	1,068	82.00	1,058	32.00	1,104	78.00
Nicotine	μg/puff	0.13	0.45	BDL	BDL	39.60	5.20	149.0	35.40	130.8	20.60	168.4	11.40	256.0	50.00
ACM	μg/puff	7.14	23.70	BDL	BDL	3,583	756	8,838	250	8,692	529.2	8,758	277.3	8,818	819.4
**Triacetin, humectants, menthol**															
Propylene glycol	μg/puff	0.24	0.80	NQ	NQ	690.0	150.0	3,760	140.0	3,880	240.0	3,860	100.0	3,980	380.0
Menthol	μg/puff	0.24	0.81	BDL	BDL	BDL	BDL	BDL	BDL	BDL	BDL	BDL	BDL	BDL	BDL
Diethylene glycol	μg/puff	0.24	0.80	BDL	BDL	BDL	BDL	BDL	BDL	BDL	BDL	BDL	BDL	BDL	BDL
Triacetin	μg/puff	0.24	0.80	BDL	BDL	BDL	BDL	BDL	BDL	BDL	BDL	BDL	BDL	BDL	BDL
Glycerol	μg/puff	1.44	4.80	NQ	NQ	1,676	392.0	2,960	200.0	3,120	220.0	2,900	120.0	2,780	320.0
Pad ethylene glycol	μg/puff	0.05	0.17	BDL	BDL	BDL	BDL	BDL	BDL	0.32	0.30	BDL	BDL	0.30	0.26
Impinger ethylene glycol	μg/puff	0.05	0.17	BDL	BDL	BDL	BDL	BDL	BDL	BDL	BDL	BDL	BDL	BDL	BDL
Pad glycidol	μg/puff	0.11	0.36	BDL	BDL	BDL	BDL	BDL	BDL	BDL	BDL	BDL	BDL	BDL	BDL
Impinger glycidol	μg/puff	0.11	0.36	BDL	BDL	BDL	BDL	BDL	BDL	BDL	BDL	BDL	BDL	BDL	BDL
**PAHs**															
Naphthalene	pg/puff	10.07	33.56	56.40	11.00	90.40	13.00	85.20	8.80	97.20	20.80	81.00	8.00	87.00	4.40
1-Methylnaphthalene	pg/puff	6.07	20.24	52.60	16.20	62.00	14.00	65.20	14.20	73.20	29.60	52.40	10.80	49.20	8.20
2-Methylnaphthalene	pg/puff	4.55	15.18	64.60	19.60	68.00	12.60	72.80	18.40	90.40	24.80	66.60	6.80	67.20	7.60
Acenaphthylene	pg/puff	4.55	15.18	23.80	6.40	28.80	9.00	24.00	4.60	19.48	2.94	NQ	NQ	NQ	NQ
Acenaphthene	pg/puff	9.60	32.00	NQ	NQ	NQ	NQ	NQ	NQ	NQ	NQ	NQ	NQ	NQ	NQ
Fluorene	pg/puff	4.72	15.72	42.20	10.00	44.20	7.00	50.20	9.20	52.40	13.00	41.20	10.60	38.00	4.40
Phenanthrene	pg/puff	3.58	11.94	262.0	20.00	262.0	18.00	286.0	28.00	274.0	34.00	260.0	18.00	256.0	16.00
Anthracene	pg/puff	4.63	15.43	18.12	3.50	23.80	4.00	26.20	4.60	20.60	3.00	24.00	4.80	NQ	NQ
Fluoranthene	pg/puff	3.94	13.13	102.4	13.40	102.4	13.00	110.8	15.20	102.8	19.80	94.20	13.80	91.20	16.00
Pyrene	pg/puff	9.43	31.44	282.0	42.00	290.0	50.00	280.0	60.00	252.0	54.00	236.0	40.00	244.0	48.00
Benzo(a)anthracene	pg/puff	7.30	24.35	NQ	NQ	NQ	NQ	NQ	NQ	NQ	NQ	BDL	BDL	BDL	BDL
Chrysene	pg/puff	4.68	15.58	NQ	NQ	NQ	NQ	NQ	NQ	NQ	NQ	NQ	NQ	NQ	NQ
Benzo(b)fluoranthene	pg/puff	16.90	56.34	BDL	BDL	BDL	BDL	BDL	BDL	BDL	BDL	BDL	BDL	BDL	BDL
Benzo(k)fluoranthene	pg/puff	11.86	39.52	BDL	BDL	BDL	BDL	BDL	BDL	BDL	BDL	BDL	BDL	BDL	BDL
Benzo(e)pyrene	pg/puff	6.96	23.19	BDL	BDL	NQ	NQ	BDL	BDL	BDL	BDL	BDL	BDL	NQ	NQ
Benzo(a)pyrene	pg/puff	10.63	35.42	BDL	BDL	BDL	BDL	BDL	BDL	BDL	BDL	BDL	BDL	BDL	BDL
Perylene	pg/puff	11.36	37.86	BDL	BDL	BDL	BDL	BDL	BDL	BDL	BDL	BDL	BDL	BDL	BDL
Indeno(1,2,3-cd)pyrene	pg/puff	10.12	33.73	BDL	BDL	NQ	NQ	BDL	BDL	BDL	BDL	BDL	BDL	BDL	BDL
Dibenz(a,h)anthracene	pg/puff	12.39	41.31	BDL	BDL	BDL	BDL	BDL	BDL	BDL	BDL	BDL	BDL	BDL	BDL
Benzo(g,h,i)perylene	pg/puff	10.12	33.73	NQ	NQ	NQ	NQ	NQ	NQ	NQ	NQ	BDL	BDL	NQ	NQ
Benzo(c)phenanthrene	pg/puff	5.38	17.92	NQ	NQ	NQ	NQ	NQ	NQ	NQ	NQ	NQ	NQ	NQ	NQ
Cyclopenta(c,d)pyrene	pg/puff	8.11	27.03	NQ	NQ	BDL	BDL	BDL	BDL	BDL	BDL	BDL	BDL	BDL	BDL
Benzo(j)aceanthrylene	pg/puff	10.37	34.56	BDL	BDL	BDL	BDL	BDL	BDL	BDL	BDL	BDL	BDL	BDL	BDL
**Volatiles**															
1,3-Butadiene	ng/puff	5.70	19.01	BDL	BDL	BDL	BDL	BDL	BDL	BDL	BDL	BDL	BDL	BDL	BDL
Isoprene	ng/puff	8.12	27.06	BDL	BDL	BDL	BDL	BDL	BDL	BDL	BDL	BDL	BDL	BDL	BDL
Acrylonitrile	ng/puff	6.40	21.34	BDL	BDL	BDL	BDL	BDL	BDL	BDL	BDL	BDL	BDL	BDL	BDL
Benzene	ng/puff	3.41	11.37	BDL	BDL	BDL	BDL	BDL	BDL	BDL	BDL	BDL	BDL	BDL	BDL
Toluene	ng/puff	12.23	40.78	BDL	BDL	BDL	BDL	BDL	BDL	BDL	BDL	BDL	BDL	BDL	BDL
Ethylbenzene	ng/puff	2.88	9.61	BDL	BDL	BDL	BDL	BDL	BDL	BDL	BDL	BDL	BDL	BDL	BDL
Ethylene oxide	ng/puff	7.18	23.98	BDL	BDL	BDL	BDL	BDL	BDL	BDL	BDL	BDL	BDL	BDL	BDL
Vinyl chloride	pg/puff	131.5	438.3	BDL	BDL	BDL	BDL	BDL	BDL	BDL	BDL	BDL	BDL	BDL	BDL
Propylene oxide	ng/puff	3.12	10.40	BDL	BDL	BDL	BDL	BDL	BDL	BDL	BDL	BDL	BDL	BDL	BDL
Furan	ng/puff	5.63	18.75	BDL	BDL	BDL	BDL	BDL	BDL	BDL	BDL	BDL	BDL	BDL	BDL
Vinyl acetate	ng/puff	2.19	7.29	BDL	BDL	BDL	BDL	BDL	BDL	BDL	BDL	BDL	BDL	BDL	BDL
Nitromethane	ng/puff	1.70	5.66	BDL	BDL	BDL	BDL	BDL	BDL	BDL	BDL	BDL	BDL	BDL	BDL
**Tobacco-specific nitrosamines**															
NNN	pg/puff	9.85	32.82	BDL	BDL	BDL	BDL	BDL	BDL	BDL	BDL	BDL	BDL	BDL	BDL
NAT	pg/puff	19.51	65.04	BDL	BDL	BDL	BDL	BDL	BDL	BDL	BDL	BDL	BDL	BDL	BDL
NAB	pg/puff	5.36	17.85	BDL	BDL	BDL	BDL	BDL	BDL	BDL	BDL	BDL	BDL	BDL	BDL
NNK	pg/puff	15.05	50.18	BDL	BDL	BDL	BDL	BDL	BDL	BDL	BDL	BDL	BDL	BDL	BDL
**Carbonyls**															
Formaldehyde	ng/puff	5.49	18.30	NQ	NQ	268.0	148.0	52.80	10.80	179.0	244.6	109.4	25.60	123.3	17.83
Acetaldehyde	ng/puff	9.95	33.17	NQ	NQ	230.0	134.0	NQ	NQ	100.6	169.6	NQ	NQ	34.12	7.30
Acetone	ng/puff	6.31	21.03	88.60	26.80	135.8	36.60	111.0	17.00	140.8	10.80	176.8	25.40	170.3	19.44
Propionaldehyde	ng/puff	4.84	16.13	NQ	NQ	96.20	71.00	NQ	NQ	NQ	NQ	NQ	NQ	NQ	NQ
Acrolein	ng/puff	9.28	30.92	BDL	BDL	346.0	200.0	BDL	BDL	BDL	BDL	NQ	NQ	NQ	NQ
Isobutyraldehyde	ng/puff	1.65	5.51	NQ	NQ	164.0	35.20	506.0	78.00	BDL	BDL	5.66	10.82	BDL	BDL
Methyl Ethyl Ketone	ng/puff	5.13	17.09	BDL	BDL	BDL	BDL	BDL	BDL	BDL	BDL	BDL	BDL	BDL	BDL
3-Buten-2-one	ng/puff	6.21	20.70	BDL	BDL	BDL	BDL	BDL	BDL	BDL	BDL	BDL	BDL	BDL	BDL
n-Butyraldehyde	ng/puff	3.51	11.71	BDL	BDL	BDL	BDL	BDL	BDL	BDL	BDL	BDL	BDL	BDL	BDL
Crotonaldehyde	ng/puff	6.23	20.75	BDL	BDL	BDL	BDL	BDL	BDL	BDL	BDL	BDL	BDL	BDL	BDL
Glycolaldehyde	ng/puff	7.45	24.84	BDL	BDL	60.20	39.20	NQ	NQ	35.20	22.00	33.20	6.80	BDL	BDL
Acetoin	ng/puff	6.73	22.43	BDL	BDL	BDL	BDL	BDL	BDL	BDL	BDL	BDL	BDL	BDL	BDL
Glyoxal	ng/puff	2.52	8.40	BDL	BDL	18.76	9.54	NQ	NQ	45.20	86.60	14.78	7.36	38.33	7.59
Methylglyoxal	ng/puff	1.54	5.12	BDL	BDL	73.20	34.20	36.40	11.60	135.0	163.4	83.40	19.20	145.9	20.47
2,3-Butanedione	ng/puff	1.74	5.80	BDL	BDL	NQ	NQ	BDL	BDL	BDL	BDL	BDL	BDL	BDL	BDL
2,3-Pentanedione	ng/puff	3.51	11.71	BDL	BDL	BDL	BDL	BDL	BDL	BDL	BDL	BDL	BDL	BDL	BDL
2,3-Hexanedione	ng/puff	3.81	12.71	BDL	BDL	BDL	BDL	BDL	BDL	BDL	BDL	BDL	BDL	BDL	BDL
2,3-Heptanedione	ng/puff	4.68	15.61	BDL	BDL	BDL	BDL	BDL	BDL	BDL	BDL	BDL	BDL	BDL	BDL
**Phenolic compounds**															
Hydroquinone	ng/puff	12.44	41.47	BDL	BDL	BDL	BDL	BDL	BDL	BDL	BDL	BDL	BDL	BDL	BDL
Resorcinol	ng/puff	3.29	10.98	BDL	BDL	BDL	BDL	BDL	BDL	BDL	BDL	BDL	BDL	BDL	BDL
Catechol	ng/puff	5.14	17.13	BDL	BDL	BDL	BDL	BDL	BDL	BDL	BDL	BDL	BDL	BDL	BDL
Phenol	ng/puff	5.15	17.17	BDL	BDL	BDL	BDL	BDL	BDL	BDL	BDL	BDL	BDL	BDL	BDL
*p*-Cresol	ng/puff	2.06	6.86	BDL	BDL	BDL	BDL	BDL	BDL	BDL	BDL	BDL	BDL	BDL	BDL
*m*-Cresol	ng/puff	1.13	3.77	BDL	BDL	BDL	BDL	BDL	BDL	BDL	BDL	BDL	BDL	BDL	BDL
*o-*Cresol	ng/puff	1.54	5.15	BDL	BDL	BDL	BDL	BDL	BDL	BDL	BDL	BDL	BDL	BDL	BDL
**Aromatic flavourants**															
Methyl acetate	ng/puff	72.00	240.00	BDL	BDL	BDL	BDL	BDL	BDL	BDL	BDL	BDL	BDL	BDL	BDL
Ethyl acetate	ng/puff	60.00	200.00	NQ	NQ	NQ	NQ	NQ	NQ	NQ	NQ	NQ	NQ	NQ	NQ
1-Butanol	ng/puff	60.00	200.00	BDL	BDL	BDL	BDL	BDL	BDL	BDL	BDL	BDL	BDL	BDL	BDL
Isobutyl acetate	ng/puff	60.00	200.00	BDL	BDL	BDL	BDL	BDL	BDL	BDL	BDL	BDL	BDL	BDL	BDL
Furfural	ng/puff	84.00	280.00	BDL	BDL	BDL	BDL	BDL	BDL	BDL	BDL	BDL	BDL	BDL	BDL
Isoamyl acetate	ng/puff	96.00	320.00	BDL	BDL	BDL	BDL	BDL	BDL	BDL	BDL	BDL	BDL	BDL	BDL
Benzyl acetate	ng/puff	60.00	200.00	BDL	BDL	BDL	BDL	BDL	BDL	BDL	BDL	BDL	BDL	BDL	BDL
Ethyl acetoacetate	ng/puff	4.80	16.00	BDL	BDL	BDL	BDL	BDL	BDL	BDL	BDL	BDL	BDL	BDL	BDL
**Acids**															
Acetic acid	ng/puff	284.00	946.00	BDL	BDL	BDL	BDL	BDL	BDL	BDL	BDL	BDL	BDL	BDL	BDL
Propionic acid	ng/puff	36.00	120.00	BDL	BDL	154.81	14.60	NQ	NQ	BDL	BDL	BDL	BDL	BDL	BDL

*ACM, aerosol collected matter; BDL, below detection limit; LOD, limit of detection; LOQ, limit of quantification; NAB, nitrosoanabasine; NAT, nitrosoanatabine; NNK, 4-N-nitrosomethylamino)-1-(3-pyridyl)-1-butanone; NNN, nitrosonornicotine; NQ, not quantified; PAH, polycyclic aromatic hydrocarbon. BT, Blended Tobacco, MB, MasterBlend*.

**Table 3:** Cigarette smoke emissions per-puff, and puff numbers from 1R6F and B&H Skyblue.

**Smoke constituent**	**Unit**	**Air blank**				**B+H Skyblue cigarette**				**Ky1R6F reference cigarette**			
		**LOD**	**LOQ**	**Mean**	**SD**	**LOD**	**LOQ**	**Mean**	**SD**	**LOD**	**LOQ**	**MS**	**SD**
**CO**													
Puff count	per cig			10.00	0.00			8.50	0.900			9.30	0.400
CO	mg/puff	1.59E−02	5.30E−02	NQ	NQ	1.92E−02	6.39E−02	2.765	0.153	1.64E−02	5.47E−02	2.892	0.075
**NFDPM, water, and nicotine**													
Puff count	per cig			10.00	0.00			8.30	0.300			9.70	0.400
Water	mg/puff	6.38E−03	2.13E−02	BDL	BDL	7.68E−03	2.56E−02	1.663	0.253	6.57E−03	2.56E−02	1.629	0.103
Nicotine	mg/puff	2.24E−04	7.48E−04	BDL	BDL	2.70E−04	9.02E−04	0.210	0.013	2.31E−04	9.02E−04	0.210	0.010
NFDPM	mg/puff	1.19E−02	3.95E−02	BDL	BDL	1.43E−02	4.76E−02	3.145	0.398	1.23E−02	4.07E−02	2.990	0.144
**Triacetin, humectants, menthol**													
Puff count	per cig			10.00	0.00			8.30	0.300			9.70	0.400
Propylene glycol	mg/puff	4.00E−04	1.33E−03	BDL	BDL	4.82E−04	1.61E−03	0.002	0.000	4.13E−04	1.38E−03	0.049	0.003
Menthol	mg/puff	4.07E−04	1.36E−03	BDL	BDL	4.90E−04	1.63E−03	BDL	BDL	4.20E−04	1.40E−03	BDL	BDL
Diethylene glycol	mg/puff	4.00E−04	1.33E−03	BDL	BDL	4.82E−04	1.61E−03	BDL	BDL	4.12E−04	1.37E−03	BDL	BDL
Triacetin	mg/puff	4.01E−04	1.34E−03	BDL	BDL	4.84E−04	1.61E−03	0.123	0.012	4.14E−04	1.38E−03	0.162	0.008
Glycerol	mg/puff	2.40E−03	7.99E−03	BDL	BDL	2.89E−03	9.63E−03	0.046	0.004	2.47E−03	8.24E−03	0.164	0.006
Pad ethylene glycol	mg/puff	8.41E−05	2.80E−04	BDL	BDL	1.01E−04	3.38E−04	BDL	BDL	8.67E−05	2.89E−04	BDL	BDL
Impinger ethylene glycol	mg/puff	8.41E−05	2.80E−04	BDL	BDL	1.01E−04	3.38E−04	BDL	BDL	8.67E−05	2.89E−04	BDL	BDL
Pad glycidol	mg/puff	1.80E−04	6.00E−04	BDL	BDL	2.17E−04	7.23E−04	0.001	0.002	1.86E−04	6.19E−04	BDL	BDL
Impinger glycidol	mg/puff	1.80E−04	6.00E−04	BDL	BDL	2.17E−04	7.23E−04	BDL	BDL	1.86E−04	6.19E−04	BDL	BDL
**PAH**													
Puff count	per cig			10.00	0.00			8.80	0.700			9.60	0.300
Naphthalene	ng/puff	1.50E−02	4.99E−02	2.26	0.55	1.91E−02	6.36E−02	173.9	13.52	1.75E−02	5.83E−02	139.3	10.83
1-Methylnaphthalene	ng/puff	9.04E−03	3.01E−02	2.66	0.53	1.15E−02	3.83E−02	126.7	8.864	1.05E−02	3.51E−02	106.6	1.146
2-Methylnaphthalene	ng/puff	6.78E−03	2.26E−02	3.26	0.67	8.62E−03	2.87E−02	132.5	9.091	7.91E−03	2.64E−02	115.5	1.458
Acenaphthylene	ng/puff	6.78E−03	2.26E−02	0.51	0.10	8.62E−03	2.87E−02	20.45	0.795	7.91E−03	2.64E−02	19.48	2.292
Acenaphthene	ng/puff	1.43E−02	4.76E−02	0.31	0.04	1.82E−02	6.06E−02	10.09	1.114	1.67E−02	5.56E−02	8.563	0.500
Fluorene	ng/puff	7.02E−03	2.34E−02	0.97	0.19	8.93E−03	2.98E−02	37.95	3.864	8.19E−03	2.73E−02	34.48	1.771
Phenanthrene	ng/puff	5.33E−03	1.78E−02	0.65	0.10	6.78E−03	2.26E−02	20.45	2.045	6.22E−03	2.07E−02	20.21	1.042
Anthracene	ng/puff	6.89E−03	2.30E−02	0.18	0.04	8.77E−03	2.92E−02	10.16	1.170	8.04E−03	2.68E−02	10.52	0.625
Fluoranthene	ng/puff	5.86E−03	1.95E−02	0.24	0.03	7.46E−03	2.49E−02	13.75	1.364	6.84E−03	2.28E−02	12.29	0.729
Pyrene	ng/puff	1.40E−02	4.68E−02	0.29	0.07	1.79E−02	5.96E−02	11.01	1.170	1.64E−02	5.46E−02	9.917	0.563
Benzo(a)anthracene	ng/puff	1.09E−02	3.62E−02	NQ	NQ	1.38E−02	4.61E−02	3.580	0.489	1.27E−02	4.23E−02	3.292	0.198
Chrysene	ng/puff	6.96E−03	2.32E−02	0.07	0.02	8.85E−03	2.95E−02	3.966	0.409	8.12E−03	2.71E−02	3.750	0.104
Benzo(b)fluoranthene	ng/puff	2.52E−02	8.38E−02	BDL	BDL	3.20E−02	1.07E−01	1.648	0.170	2.93E−02	9.78E−02	1.313	0.073
Benzo(k)fluoranthene	ng/puff	1.76E−02	5.88E−02	BDL	BDL	2.25E−02	7.48E−02	0.732	0.023	2.06E−02	6.86E−02	0.605	0.059
Benzo(e)pyrene	ng/puff	1.04E−02	3.45E−02	NQ	NQ	1.32E−02	4.39E−02	0.919	0.108	1.21E−02	4.03E−02	0.753	0.081
Benzo(a)pyrene	ng/puff	1.58E−02	5.27E−02	NQ	NQ	2.01E−02	6.71E−02	1.852	0.193	1.84E−02	6.15E−02	1.719	0.125
Perylene	ng/puff	1.69E−02	5.63E−02	BDL	BDL	2.15E−02	7.17E−02	0.289	0.058	1.97E−02	6.57E−02	0.271	0.022
Indeno(1,2,3-cd)pyrene	ng/puff	1.51E−02	5.02E−02	BDL	BDL	1.92E−02	6.39E−02	0.663	0.094	1.76E−02	5.86E−02	0.624	0.033
Dibenz(a,h)anthracene	ng/puff	1.84E−02	6.15E−02	BDL	BDL	2.35E−02	7.82E−02	0.114	0.019	2.15E−02	7.17E−02	0.114	0.024
Benzo(g,h,i)perylene	ng/puff	1.51E−02	5.02E−02	BDL	BDL	1.92E−02	6.39E−02	0.491	0.055	1.76E−02	5.86E−02	0.433	0.044
Benzo(c)phenanthrene	ng/puff	8.00E−03	2.67E−02	NQ	NQ	1.02E−02	3.39E−02	0.690	0.061	9.33E−03	3.11E−02	0.589	0.055
Cyclopenta(c,d)pyrene	ng/puff	1.21E−02	4.02E−02	BDL	BDL	1.54E−02	5.12E−02	1.580	0.182	1.41E−02	4.69E−02	1.396	0.323
Benzo(j)aceanthrylene	ng/puff	1.54E−02	5.14E−02	BDL	BDL	1.96E−02	6.55E−02	0.123	0.022	1.80E−02	6.00E−02	0.107	0.007
**Volatiles**													
Puff count	per cig			10.00	0.00			8.40	0.700			9.20	0.500
1,3-Butadiene	μg/puff	1.90E−02	6.33E−02	NQ	NQ	2.26E−02	7.54E−02	10.31	0.726	2.07E−02	6.88E−02	9.978	0.500
Isoprene	μg/puff	2.70E−02	9.01E−02	0.39	0.05	3.22E−02	1.07E−01	83.10	5.476	2.94E−02	9.80E−02	86.20	5.000
Acrylonitrile	μg/puff	2.13E−02	7.11E−02	NQ	NQ	2.54E−02	8.46E−02	2.321	0.202	2.32E−02	7.72E−02	2.478	0.283
Benzene	μg/puff	1.12E−02	3.73E−02	0.20	0.02	1.33E−02	4.44E−02	8.512	0.679	1.22E−02	4.06E−02	8.652	1.043
Toluene	μg/puff	4.08E−02	1.36E−01	0.92	0.12	4.86E−02	1.62E−01	12.38	1.429	4.43E−02	1.48E−01	13.59	1.848
Ethylbenzene	μg/puff	9.60E−03	3.20E−02	0.18	0.03	1.14E−02	3.81E−02	1.238	0.155	1.04E−02	3.48E−02	1.293	0.130
Ethylene oxide	μg/puff	2.39E−02	7.93E−02	BDL	BDL	2.85E−02	9.44E−02	1.905	0.119	2.60E−02	8.62E−02	1.946	0.228
Vinyl chloride	ng/puff	4.38E−01	1.46E+00	BDL	BDL	5.21E−01	1.74E+00	9.595	1.155	4.76E−01	1.59E+00	11.09	0.543
Propylene oxide	ng/puff	1.04E+01	3.47E+01	BDL	BDL	1.24E+01	4.13E+01	115.1	8.095	1.13E+01	3.77E+01	215.3	13.26
Furan	μg/puff	1.87E−02	6.27E−02	NQ	NQ	2.22E−02	7.46E−02	6.155	0.702	2.03E−02	6.81E−02	5.989	0.696
Vinyl acetate	ng/puff	7.30E+00	2.43E+01	BDL	BDL	8.69E+00	2.89E+01	77.50	12.14	7.93E+00	2.64E+01	62.39	7.174
Nitromethane	ng/puff	5.67E+00	1.89E+01	BDL	BDL	6.75E+00	2.25E+01	27.98	4.048	6.16E+00	2.05E+01	49.46	9.565
**Tobacco–specific nitrosamines**													
Puff count	per cig			10.00	0.00			8.60	0.800			9.30	0.400
NNN	ng/puff	1.64E−02	5.47E−02	BDL	BDL	1.91E−02	6.36E−02	9.105	2.116	1.76E−02	5.88E−02	22.69	1.398
NAT	ng/puff	3.25E−02	1.08E−01	BDL	BDL	3.78E−02	1.26E−01	17.79	3.488	3.50E−02	1.17E−01	26.13	1.828
NAB	ng/puff	8.93E−03	2.98E−02	BDL	BDL	1.04E−02	3.46E−02	2.186	0.407	9.60E−03	3.20E−02	2.667	0.344
NNK	ng/puff	2.51E−02	8.36E−02	BDL	BDL	2.92E−02	9.73E−02	9.093	2.023	2.70E−02	8.99E−02	20.97	1.075
**Carbonyls**													
Puff count	per cig			10.00	0.00			8.10	1.000			9.10	0.500
Formaldehyde	μg/puff	1.37E−01	4.57E−01	NQ	NQ	1.69E−01	5.65E−01	5.235	0.852	1.51E−01	5.03E−01	4.879	0.319
Acetaldehyde	μg/puff	2.49E−01	8.29E−01	NQ	NQ	3.07E−01	1.02E+00	177.4	16.91	2.73E−01	9.11E−01	158.9	5.385
Acetone	μg/puff	1.58E−01	5.26E−01	BDL	BDL	1.95E−01	6.49E−01	65.68	6.667	1.73E−01	5.78E−01	62.31	3.187
Propionaldehyde	μg/puff	1.21E−01	4.03E−01	BDL	BDL	1.49E−01	4.98E−01	15.43	1.728	1.33E−01	4.43E−01	13.74	1.319
Acrolein	μg/puff	2.32E−01	7.73E−01	BDL	BDL	2.86E−01	9.54E−01	15.93	1.358	2.55E−01	8.49E−01	14.51	1.099
Isobutyraldehyde	μg/puff	4.13E−02	1.38E−01	BDL	BDL	5.10E−02	1.70E−01	6.272	0.815	4.54E−02	1.51E−01	5.000	0.747
Methyl ethyl ketone	μg/puff	1.28E−01	4.27E−01	NQ	NQ	1.58E−01	5.28E−01	17.41	1.605	1.41E−01	4.70E−01	15.93	0.769
3-Buten-2-one	μg/puff	1.55E−01	5.17E−01	BDL	BDL	1.92E−01	6.39E−01	7.988	0.741	1.71E−01	5.69E−01	7.462	0.385
n-Butyraldehyde	μg/puff	8.78E−02	2.93E−01	BDL	BDL	1.08E−01	3.61E−01	4.469	0.506	9.65E−02	3.22E−01	3.802	0.352
Crotonaldehyde	μg/puff	1.56E−01	5.19E−01	BDL	BDL	1.92E−01	6.41E−01	5.321	0.654	1.71E−01	5.70E−01	4.484	0.242
Glycolaldehyde	μg/puff	1.86E−01	6.21E−01	BDL	BDL	2.30E−01	7.67E−01	7.222	0.778	2.05E−01	6.82E−01	6.220	0.934
Acetoin	μg/puff	1.68E−01	5.61E−01	BDL	BDL	2.08E−01	6.92E−01	2.864	0.321	1.85E−01	6.16E−01	1.495	0.176
Glyoxal	μg/puff	6.30E−02	2.10E−01	BDL	BDL	7.78E−02	2.59E−01	0.631	0.099	6.92E−02	2.31E−01	0.897	0.148
Methylglyoxal	μg/puff	3.84E−02	1.28E−01	BDL	BDL	4.74E−02	1.58E−01	1.840	0.198	4.22E−02	1.41E−01	1.868	0.154
2,3-Butanedione	μg/puff	4.35E−02	1.45E−01	0.24	0.17	5.37E−02	1.79E−01	19.01	0.988	4.78E−02	1.59E−01	17.58	0.879
2,3-Pentanedione	μg/puff	8.78E−02	2.93E−01	NQ	NQ	1.08E−01	3.61E−01	3.321	0.222	9.65E−02	3.22E−01	2.813	0.121
2,3-Hexanedione	μg/puff	9.54E−02	3.18E−01	BDL	BDL	1.18E−01	3.92E−01	NQ	NQ	1.05E−01	3.49E−01	NQ	NQ
2,3-Heptanedione	μg/puff	1.17E−01	3.90E−01	BDL	BDL	1.45E−01	4.82E−01	BDL	BDL	1.29E−01	4.29E−01	BDL	BDL
**Phenolic compounds**													
Puff count	per cig			10.00	0.00			8.13	0.365			9.42	0.512
Hydroquinone	μg/puff	1.35E−01	4.51E−01	BDL	BDL	1.67E−01	5.55E−01	14.939	0.467	1.44E−01	4.79E−01	11.78	0.818
Resorcinol	μg/puff	3.95E−02	1.32E−01	BDL	BDL	4.85E−02	1.62E−01	0.343	0.054	4.19E−02	1.40E−01	0.308	0.061
Catechol	μg/puff	1.21E−01	4.03E−01	BDL	BDL	1.49E−01	4.96E−01	15.519	0.466	1.28E−01	4.28E−01	11.82	0.644
Phenol	μg/puff	1.43E−01	4.78E−01	BDL	BDL	1.76E−01	5.88E−01	3.276	0.165	1.52E−01	5.07E−01	1.595	0.218
*p*-Cresol	μg/puff	2.07E−02	6.91E−02	BDL	BDL	2.55E−02	8.50E−02	1.526	0.087	2.20E−02	7.34E−02	0.865	0.095
*m*-Cresol	μg/puff	4.51E−02	1.50E−01	BDL	BDL	5.55E−02	1.85E−01	0.634	0.034	4.79E−02	1.60E−01	0.360	0.042
*o*-Cresol	μg/puff	1.84E−02	6.14E−02	BDL	BDL	2.26E−02	7.55E−02	0.792	0.049	1.95E−02	6.51E−02	0.409	0.046

*NFDPM, nicotine-free dry particulate matter; BDL, below detection limit; LOD, limit of detection; LOQ, limit of quantification; NAB, nitrosoanabasine; NAT, nitrosoanatabine; NNK, 4-N-nitrosomethylamino)-1-(3-pyridyl)-1-butanone; NNN, nitrosonornicotine; NQ, not quantified; PAH, polycyclic aromatic hydrocarbon*.

**Table 4:** Toxicant to nicotine ratios calculated for the analytes providing quantifiable values from the e-cigarettes in this study.

**Parameter per mg nicotine**	**ePen2 18 BT**		**ePen3 18 BT**		**ePen3 MB 12 Low BA**		**ePen3 MB 18 Med. BA**		**ePen3 MB 30 High BA**		**Ky1R6F**		**B&H Skyblue**	
	**mean**	**SD**	**mean**	**SD**	**mean**	**SD**	**mean**	**SD**	**mean**	**SD**	**mean**	**SD**	**mean**	**SD**
Water (mg)	25.66	3.6	7.31	0.24	8.16	0.63	6.29	0.19	4.31	0.30	7.75	0.56	7.92	1.11
ACM/NFDPM (mg)	63.99	15.46	51.00	1.44	57.31	3.37	44.72	1.43	29.14	2.76	14.22	0.92	14.97	1.76
Propylene glycol (mg)	17.44	3.78	25.28	0.90	29.71	1.83	22.97	0.62	15.54	1.51	0.23	0.01	0.01	0.00
Glycerol (mg)	42.38	9.94	19.82	1.33	23.79	1.73	17.26	0.71	10.85	1.22	0.78	0.01	0.22	0.02
Pad Ethylene glycol (mg)	BDL	BDL	BDL	BDL	0.002	0.002	BDL	BDL	0.001	0.001	BDL	BDL	BDL	BDL
Naphthalene (ng)	2.29	0.33	0.57	0.06	0.74	0.16	0.48	0.05	0.34	0.02	660.85	65.14	829.71	88.12
1-Methylnaphthalene (ng)	1.57	0.35	0.44	0.10	0.56	0.23	0.31	0.06	0.19	0.03	505.17	14.33	603.98	51.14
2-Methylnaphthalene (ng)	1.72	0.32	0.49	0.12	0.69	0.19	0.40	0.04	0.26	0.03	547.74	17.47	631.68	54.72
Acenaphthylene (ng)	0.73	0.23	0.16	0.03	0.15	0.02	NQ	NQ	NQ	NQ	92.52	11.92	97.53	9.87
Fluorene (ng)	1.12	0.18	0.34	0.06	0.40	0.10	0.24	0.06	0.15	0.02	163.53	7.64	180.56	19.22
Phenanthrene (ng)	6.62	0.47	1.92	0.19	2.10	0.26	1.54	0.11	1.00	0.07	95.92	5.19	97.68	11.48
Anthracene (ng)	0.60	0.10	0.18	0.03	0.16	0.02	0.14	0.03	NQ	NQ	49.82	2.75	48.46	6.33
Fluoranthene (ng)	2.59	0.33	0.74	0.10	0.79	0.15	0.56	0.08	0.36	0.06	58.48	3.81	65.83	8.46
Pyrene (ng)	7.31	1.27	1.88	0.40	1.93	0.24	1.41	0.19	0.96	0.19	47.01	2.90	52.55	7.08
Formaldehyde (ug)	6.80	3.73	0.35	0.07	1.37	1.87	0.65	0.15	0.48	0.07	23.16	2.22	25.37	5.75
Acetaldehyde (ug)	5.83	3.38	NQ	NQ	0.77	1.30	NQ	NQ	0.13	0.03	753.87	41.62	852.17	62.13
Acetone (ug)	3.44	0.93	0.74	0.11	1.08	0.08	1.05	0.15	0.67	0.08	295.48	18.21	315.00	18.88
Propionaldehyde (ug)	2.43	1.79	NQ	NQ	NQ	NQ	NQ	NQ	NQ	NQ	64.95	5.26	73.84	1.93
Acrolein (ug)	8.74	5.04	BDL	BDL	BDL	BDL	NQ	NQ	NQ	NQ	68.68	4.52	76.76	4.14
Isobutyraldehyde (ug)	4.15	0.89	3.40	0.52	BDL	BDL	0.03	0.06	BDL	BDL	23.64	3.03	29.97	0.91
Glycoaldehyde (ug)	1.52	0.99	NQ	NQ	0.27	0.17	0.20	0.04	BDL	BDL	29.60	5.49	34.92	4.99
Glyoxal (ug)	0.47	0.24	NQ	NQ	0.35	0.66	0.09	0.04	0.15	0.03	4.24	0.61	3.03	0.45
Methylglyoxal (ug)	1.85	0.86	0.24	0.08	1.03	1.25	0.49	0.11	0.57	0.08	8.88	0.67	8.97	1.83

*NQ, not quantified; BDL, below detection limit*.

**Table 5:** Per-puff metals emission data from e-cigarettes, obtained after accelerated aging at 40°C/75% RH for 3 months, and tobacco reference cigarette.

		**Air/method blank and vapor**		**Air/method blank values**		**ePen3 18 BT**		**ePen3 MB 12 Low BA**		**ePen3 MB 18 Medium BA**		**ePen3 MB 30 High BA**		**Ky1R6F reference cigarette**		**Ky1R6F reference cigarette**	
**Aerosol/Smoke constituent**	**Unit**	**LOD**	**LOQ**	**Mean**	**SD**	**Mean**	**SD**	**Mean**	**SD**	**Mean**	**SD**	**Mean**	**SD**	**LOD**	**LOQ**	**Mean**	**SD**
Puff count						25		25		25		25				8.80	0.20
**Coil metals**																	
Nickel	ng/puff	0.25	2.17	NQ	NQ	NQ	NQ	BDL	BDL	NQ	NQ	NQ	NQ	0.32	1.08	NQ	NQ
Iron	ng/puff	0.33	1.09	3.55	1.43	2.71	0.93	1.30	0.42	1.94	0.81	4.58	0.68	0.64	2.13	4.05	0.53
**Other metals**																	
Aluminum	ng/puff	0.39	1.29	4.15	3.02	NR	NR	7.66	1.28	8.13	1.06	3.36	0.39	NR	NR	NR	NR
Arsenic	ng/puff	0.07	0.23	NQ	NQ	BDL	BDL	BDL	BDL	BDL	BDL	NR	NR	0.10	0.33	0.86	0.02
Cadmium	ng/puff	0.04	0.14	NQ	NQ	BDL	BDL	BDL	BDL	BDL	BDL	BDL	BDL	0.19	0.62	10.12	0.15
Chromium	ng/puff	0.06	0.19	1.62	0.81	1.81	0.33	1.19	0.21	1.16	0.14	1.54	0.36	0.15	0.51	NQ	NQ
Copper	ng/puff	0.18	0.60	NQ	NQ	BDL	BDL	BDL	BDL	NQ	NQ	NQ	NQ	0.28	0.93	3.49	0.20
Lead	ng/puff	0.03	0.11	NQ	NQ	NQ	NQ	BDL	BDL	0.13	0.17	0.12	0.04	0.52	1.74	3.20	0.10
Manganese	ng/puff	0.23	0.76	NQ	NQ	BDL	BDL	NQ	NQ	NQ	NQ	NQ	NQ	NR	NR	NR	NR
Molybdenum	ng/puff	0.11	0.36	0.54	0.38	0.44	0.17	0.55	0.20	0.37	0.09	NQ	NQ	NR	NR	NR	NR
Zinc	ng/puff	0.70	2.34	2.30	0.87	NQ	NQ	NQ	NQ	3.34	0.39	9.18	2.54	2.00	6.70	38.0	1.00
**Mercury**																	
Puff Count						50		25		25		25				10.50	0.60
Mercury	ng/puff	0.04	0.14	BDL	BDL	BDL	BDL	NQ	NQ	NQ	NQ	NQ	NQ	0.08	0.27	0.37	0.04

*NR, not reported; NQ, not quantified; BDL, below detection limit*.

### Nicotine, Aerosol Mass, CO, and Water Emissions

The nicotine per-puff yields from the ePen3 samples were 3–7 times higher as compared with ePen2, depending on the nicotine concentration of the e-liquid. Nicotine per-puff emissions from ePen2 were 81% lower than those from both cigarettes. Due to the different nicotine concentrations of the ePen3 e-liquids, the percentage difference in nicotine emissions between the ePen3 samples and the two cigarettes varied from 38% lower to 22% higher.

Aerosol collected matter (ACM) per puff was, on average, 2.4 times higher from the ePen3 aerosol samples than from ePen2. The per-puff ACM yield from ePen2 was 15–20% higher than the cigarette tar yield. In contrast, the per-puff ACM yields from all e-Pen3 variants were 176–196% higher. ePen2 per-nicotine ACM yields were significantly higher than from the ePen3 samples with at least 18 mg/mL nicotine. The cigarette per-nicotine emissions were not significantly different from each other, but were significantly lower than the corresponding ACM emissions from all of the e-cigarettes.

The CO emissions from all e-cigarettes were below the detection limit (BDL) and therefore >99% lower than those from either cigarette. The air/method background values for this group of analytes were all BDL.

Water emissions per-puff were comparable among all e-cigarette samples. The per-puff water emissions from all five e-cigarettes were consistently 32–39% lower than those from the two cigarettes. Per-nicotine water emissions from the ePen2 sample were significantly higher than from the ePen3 samples due to the lower nicotine emission from ePen2. Per-nicotine water emissions from the two combustible cigarettes were not significantly different from each other, but were significantly lower than from ePen2 and higher than ePen3 30 mg/mL.

### Triacetin, Humectants, Menthol

Air/method background levels of menthol, diethylene glycol, triacetin, ethylene glycol, and glycidol were all BDL. Background levels of PG and VG were detected but too low to quantify (i.e., <LOQ), which may reflect ambient contamination from repeated device testing in the e-cigarette laboratory.

Emissions of menthol, diethylene glycol, triacetin, and glycidol were BDL for all five e-cigarettes. Ethylene glycol emissions were quantifiable from two e-cigarettes but BDL with the other samples.

All e-cigarette aerosols contained considerable quantities of PG and VG. Per-puff emissions of PG were 6 times higher from the ePen3 samples than from ePen2, reflecting both the higher proportion of PG in the ePen3 e-liquids and the 2–3-fold higher per-puff ACM from ePen3 samples as compared with ePen2. Per-nicotine PG emissions from the two combustible cigarettes were not significantly different from each other, but were significantly lower than those from any of the e-cigarettes. ePen2 PG/nicotine was significantly lower than from all ePen3 variants, except for ePen3 30 mg/mL high BA.

In comparison to the B&H Skyblue cigarette, per-puff VG emissions from the five e-cigarettes were between 3,500 and 6,750% higher, and PG emissions were between 32,000 and 183,000% higher. VG and PG emissions were also higher from the e-cigarettes than from the 1R6F cigarette, but to a lesser degree: VG emissions were 900–1,800% higher and PG emissions were 1,300–8,000% higher. Per-nicotine VG emissions from the two combustible cigarettes were not significantly different from each other, but were significantly lower than from any of the e-cigarettes tested in this study. ePen2 VG/nicotine was significantly higher than from all ePen3 variants. Per-nicotine VG from the ePen3 products with a nicotine loading below 30 mg/mL were statistically equivalent.

Glycidol was not detected in any of the e-cigarette aerosols, but was quantified in B&H Skyblue cigarette smoke but not in 1R6F smoke. The per-puff emissions from the e-cigarettes were at least 95% lower than those from the B&H Skyblue cigarette. The relative emissions of diethylene glycol and ethylene glycol from e-cigarettes and cigarettes could not be calculated because these analytes were not detected in sufficient numbers of samples.

### Polycyclic Aromatic Hydrocarbons

Among the 23 PAHs analyzed, 18 were either not detected in the e-cigarette aerosols or detected at extremely low levels not significantly different to the air/method blank, indicating that these compounds are not generated by the five e-cigarettes tested. For example, benzo(b)fluoranthene, benzo(k)fluoranthene, benzo(a)pyrene, perylene, dibenz(a,h)anthracene and benzo(j)aceanthrylene were BDL for all air/method blanks and e-cigarette samples. Cyclopenta(c,d)pyrene was <LOQ in the air/method blank, but not detected in any of the e-cigarette samples. Benzo(a)anthracene was <LOQ for air/method blank and three e-cigarette samples, and BDL for two of the ePen3 samples. Benzo(g,h,i)perylene was also <LOQ for air/method blank and four e-cigarette samples, but BDL for one ePen3 sample. Benzo(c)phenanthrene, acenaphthene and chrysene were <LOQ for all tested samples. 1-methylnaphthalene, 2-methylnaphthalene, fluorene, acenaphthylene, phenanthrene, fluoranthene, and pyrene were quantified in most or all samples, including the air/method blank, but their levels did not differ significantly between the e-cigarette samples and the air/method blank sample.

The per-puff levels of four PAHs were higher in e-cigarette aerosols than in air/method blanks. Indeno(1,2,3-cd)pyrene and benzo(e)pyrene were BDL for the air/method blank and almost all e-cigarette samples, but indeno(1,2,3-cd)pyrene was <LOQ for ePen2 and benzo(e)pyrene was <LOQ for ePen2 and one ePen3 sample. Anthracene emissions from ePen3 (18 mg/mL, BT) were significantly higher than the air/method blank (*p* <0.05); all other e-cigarette aerosols were not significantly different to the air/method blank. Naphthalene was significantly (up to 70%) higher in all five e-cigarette aerosols than in the air/method blank (*p* < 0.005).

Overall, on a per-puff basis, levels of PAHs were significantly higher in cigarette smoke than in the e-cigarette aerosols. Across all PAHs and e-cigarettes, per-puff levels were, on average, 98.8% lower in e-cigarette aerosol than in smoke from B&H Skyblue (range 94.5% [dibenz(a,h)anthracene] to >99.9% [multiple PAHs]). Similarly, per-puff PAH levels were, on average, 98.7% lower in aerosol from e-cigarettes than in smoke from 1R6F (range 94.5–99.9%). Expressed as a ratio to nicotine all of the PAH emissions from the e-cigarettes were substantially lower (mean 98.7%, range 84% with pyrene to >99.9% for multiple PAHs) than from both combustible cigarettes. Quantifiable per-nicotine PAH emissions had a tendency to decrease across the e-cigarettes as nicotine emissions increased (i.e., from ePen2 to increasing ePen3 nicotine content), but differences between ePen2 and ePen3 were not always significant.

### Volatile Compounds

None of the volatile organic toxicants examined were detected in the air/method blank or e-cigarette aerosols; all measurements were BDL for the five test products. In contrast, quantifiable levels of all volatile toxicants were detected in smoke from the two tobacco cigarettes. Consequently, the levels of these compounds in the aerosols from the e-cigarettes were, on average, 99.4% lower than those from the B&H Skyblue cigarette on a per-puff basis (range 97–>99.9%), and 99.6% lower than those from 1R6F (range 98.3–>99.9%).

### Tobacco-Specific Nitrosamines

Tobacco-specific nitrosamines (TSNAs) emissions both in the air/method sample and all e-cigarette aerosols were BDL. By contrast, all four TSNAs were quantified in the smoke from 1R6F and B&H Skyblue cigarettes. Emissions of TSNAs from all e-cigarette samples were therefore ~99.9% lower than those from the two tobacco cigarettes.

### Carbonyls and Dicarbonyls

Among 18 carbonyls evaluated, emissions of methyl ethyl ketone, 3-buten-2-one, *n*-butyraldehyde, crotonaldehyde, acetoin, and 2,3-pentanedione were BDL for the air/method blank and all e-cigarette samples. These six compounds were quantified in both cigarette smoke samples, and thus their levels were, on average, >99.9% lower in e-cigarette aerosols than in cigarette smoke.

2,3-Heptanedione was not detected in e-cigarette aerosols, the air/method blank, or the cigarette smoke samples. 2,3-Hexanedione was detected but not quantifiable in the cigarette samples, and not detected in any of the other samples.

Formaldehyde was not quantifiable in the air/method blank, but was quantified in all e-cigarette aerosol samples. Formaldehyde levels per-puff were higher in the ePen2 than in the ePen3 aerosol samples (*p* = 0.03), but were much higher in the two cigarette smoke samples. In comparison to B&H Skyblue cigarette smoke, levels of formaldehyde were, on average, 97.2% lower in the e-cigarette aerosols (range 94.9–99%). Similarly, the e-cigarettes had, on average, 97% lower formaldehyde emissions as compared with 1R6F (range 94.5–98.9%). Per-nicotine emissions from the two combustible cigarettes were not significantly different from each other, but were significantly higher than from any of the e-cigarettes tested in this study. Per-nicotine formaldehyde emissions from ePen2 were significantly higher than from all ePen3 samples other than the 12 mg/mL low BA sample. All ePen3 variants were not statistically different from each other.

Acetaldehyde was not quantifiable in the air/method blank or two e-cigarette samples (ePen3 [18 mg/mL, BT] and ePen3 [18 mg/mL, Medium BA]), but was quantified in aerosol from ePen2, ePen3 (12 mg/mL, Low BA) and ePen3 (30 mg/mL, High BA). Both per-puff and per-nicotine levels were significantly higher (*p* < 0.05) in the ePen2 sample than in all ePen3 samples except for ePen3 (12 mg/mL, Low BA), where high levels of variance were observed. The cigarette smoke samples contained substantially higher levels of acetaldehyde than any other carbonyl, and the acetaldehyde content of the e-cigarette aerosols was >99.9 lower than the smoke from both combustible cigarettes on both a per-puff and per-nicotine basis.

Acetone was quantified in the air/blank samples and in all e-cigarette aerosols. Acetone emissions were higher in the e-cigarettes than the air/method blank for ePen2 and most ePen3 samples (*p* < 0.05), although emissions from the ePen3 BT (18 mg/mL) sample were not significantly different from the air/method blank (*p* > 0.05). Per-puff emissions from ePen3 BT 18 mg/mLwere lower than from those of the other ePen3 samples (*p* < 0.05) except for ePen3 (12 mg/mL Low BA). On a per-nicotine basis the e-cigarette acetone emissions were not significantly different to each other, but were significantly lower than those from both combustible cigarettes (which were not significantly different to each other). In comparison to cigarette smoke, acetone emissions from the e-cigarettes were, on average, 99.6–99.8% lower than those from B&H Skyblue and 1R6F cigarette smoke on a per nicotine or per-puff basis, respectively.

Propionaldehyde was detected but not quantifiable in the air/method blank or ePen3 aerosol samples, but was quantified in the ePen2 sample. On average, propionaldehyde emissions were 99.8% lower from the e-cigarettes than from the two tobacco cigarettes.

Acrolein was not detected in the air/method blank or two of the ePen3 aerosol samples. The other two ePen3 samples showed non-quantifiable levels. The ePen2 aerosol had substantially higher and quantifiable (albeit variable) levels of acrolein than the ePen3 samples (both per-puff and per-nicotine). B&H Skyblue acrolein emissions were significantly higher than from 1R6F; both cigarette smoke emissions were significantly higher than from the e-cigarettes. Acrolein per-puff emissions were 98.2% lower (88% on a per-nicotine basis) from ePen2 than from cigarette smoke; on average, ePen3 samples were >99.9% lower from than from cigarette smoke.

Isobutyraldehyde was detected but not quantified in the air/method blank. Regarding the e-cigarettes, it was not detected in two ePen3 samples, but was quantified in the emissions of the other two ePen3 samples and ePen2. Isobutyraldehyde levels per-puff were significantly higher in emissions from ePen3 (18 mg/mL, BT) than in those from ePen2 (18 mg/mL, BT), which were in turn significantly higher than those from the other ePen3 samples. Per nicotine emissions from ePen2 and ePen3 18 mg BT were significantly higher than from the other e-cigarettes. Per nicotine emissions from B&H were significantly higher than from 1R6F. In comparison to cigarette smoke, e-Pen2 isobutyraldehyde emissions were an average of 97% lower per-puff and 84% lower per-nicotine, and ePen3 emissions were 91–99.9% lower per-puff and 87–99.9% lower per-nicotine.

Glycolaldehyde was not detected in the air/method blank, but was detected in most of the e-cigarette aerosol samples. Levels were generally higher from ePen2 than from the ePen3 samples. Glycolaldehyde was not detected in one ePen3 sample and <LOQ in another; the other two ePen3 samples had quantifiable levels that were not significantly lower than those of the ePen2 sample (*p* > 0.05). E-cigarette emissions of glycolaldehyde were, on average, 99.5% lower as compared with cigarette smoke.

Glyoxal and methylglyoxal were not detected in the air/method blank, but were detected at quantifiable levels in all e-cigarette aerosol samples except for ePen3 (18 mg/mL, BT) aerosol, where glyoxal was detected but not quantifiable. Quantifiable glyoxal emissions from the e-cigarettes were not significantly different to each other. Methyl glyoxal emissions were higher (although not statistically significant) from ePen3 (30 mg/mL, High BA) than from ePen2, ePen3 (18 mg/mL, BT), or ePen3 (18 mg/mL, Medium BA) samples (*p* > 0.05). Methyl glyoxal emissions from ePen3 (18 mg/mL, Medium BA) were higher than those from the ePen3 (18 mg/mL, BT) sample, but not statistically significant (*p* > 0.05). However, methylglyoxal emissions did not differ significantly between ePen2 and ePen3 (18 mg/mL, BT). Relative to cigarette smoke, glyoxal levels from e-cigarettes were, on average, 96.1% lower than those from B&H Skyblue (range 92.8–99.1%) and 97.3% lower than those from 1R6F (range 95–99.4%). Methylglyoxal levels were, on average, 94.9% lower from e-cigarettes than from either tobacco cigarette (range 92.1–98.0%).

2,3-Butanedione (diacetyl) was not detected in the air/method blank or in any sample other than the ePen2 aerosol, where it was not quantifiable. Diacetyl emissions from the e-cigarettes were, on average, >99.9% lower than those from the two cigarettes.

### Phenolic Compounds

None of the seven phenols measured were detected in the air/method blank, or in any of the e-cigarette aerosol samples (all BDL). By contrast, phenols were quantified in both cigarette smoke samples. Consequently, levels of the phenols in the e-cigarette aerosols were, on average, 99.8% lower than those in cigarette smoke (range 99.5–>99.9%).

### Aromatic Flavourants

Among the 10 flavourants tested, methyl acetate, 1-butanol, isobutyl acetate, furfural, isoamyl acetate, benzyl acetate, ethyl acetoacetate, and acetic acid were not detected in any of the e-cigarette aerosols or the air/method blank (all BDL). Ethyl acetate was detected, but not quantified in the air/method blank and all e-cigarette aerosol samples. Propionic acid was not detected in the air/method blank or in most of the e-cigarette samples; however, it was detected at sub-quantifiable levels in the ePen3 (18 mg/mL, BT) aerosol and at quantifiable and substantially higher levels in the ePen2 aerosol.

### Metals

Metals emissions from the e-cigarettes were measured after the cartridges containing e-liquids were stored at 40°C/75%RH for 3 months, in an accelerated aging test. The data from this exercise are presented in Table 5.

Of particular interest are the e-cigarette emissions of Ni and Fe, as they constitute the major components of the coil. The data in Table 5 show nickel emissions are <LOQ for all samples, including cigarette smoke. With the iron emissions, the e-cigarette samples were not significantly different from the air/method blank values or the cigarette smoke iron emission.

Of the other metals examined, with aluminum and molybdenum the e-cigarette emissions were not significantly different to the air/method blank values; cigarette smoke emissions were not measured for these metals. Arsenic, copper and mercury cigarette smoke emissions were quantifiable, whereas all e-cigarette emissions were <LOQ or <LOD. Manganese emissions from the e-cigarettes were also <LOQ or <LOD but the cigarette smoke emissions were not measured. Chromium e-cigarette emissions were not significantly different to the air/method blank, which was higher than the cigarette smoke emission level. Cadmium cigarette smoke emissions were 10 ng/puff, but all e-cigarette emissions were <LOD. Lead emissions from two e-cigarettes were quantifiable, but not significantly different to the air/method blank values; cigarette smoke emissions were 25 times higher. Zinc emissions from the 30 mg/mL nicotine high BA sample were significantly higher than from the other e-cigarettes which were not significantly different to the air/method blank level or <LOQ; cigarette smoke emissions were four times higher than from the high BA e-cigarette emission.

## Discussion

In this study, we quantified 97 analyte emissions from five e-cigarettes, and 84 analyte emissions from two tobacco cigarettes. Some of these analytes, including many of the additional HPHCs recently proposed by the FDA, have not previously been quantified in e-cigarette aerosols to our knowledge.

### Relevance of Air/Method Blank Measurements to E-Cigarette Emissions Testing

Recent studies have demonstrated the importance of recording baseline measurements to check for contamination when quantifying low-level emissions from e-cigarettes (Tayyarah and Long, 2014; Margham et al., 2016; Wagner et al., 2018). In particular, Margham et al. (2016) demonstrated that contamination from laboratory air and analytical methodology equipment and reagents can lead to background “blank sample” levels of some toxicants that are statistically indistinguishable from those measured in e-cigarette emissions. Such artifacts severely confound both the identification and accurate quantification of e-cigarette aerosol constituents. It is therefore essential to follow basic scientific good practice by conducting measurements of background air/method samples under identical conditions to those used for e-cigarette aerosol measurements if accurate data are sought.

As compared with a previous study in the same laboratory (Margham et al., 2016), the present air/method blank samples showed lower levels of artifacts. Some of the reduction in contaminants is down to the lower number of puffs in the current study (*n* = 50 vs. *n* = 100), which would halve the levels of contaminants per collection, but it is also the result of ongoing improvements in methodology and control of experimental protocols by the measurement laboratory. Progress in these areas does not remove the need for air/method background measurements, as demonstrated in particular by several of the individual PAH measurements. Our previous recommendation to conduct background measurements alongside e-cigarette measurements remains as pertinent today as in earlier investigations (Margham et al., 2016).

### Impact of Benzoic Acid on Aerosol Emissions

In examining the stability of BA in e-cigarettes, the focus of our investigation was the aromatic species benzene and phenol, both of which can be formed by decarboxylation reactions at temperatures of 500°C and above. Our results showed that neither benzene nor phenol was present in any of the five e-cigarette aerosols, independent of the presence or absence of BA. Similarly, the presence or absence of BA did not affect the levels of larger aromatics (PAHs) or smaller volatile hydrocarbons. Significant differences in some carbonyl emissions were observed between ePen3 (18 mg/mL, BT) and ePen3 (18 mg/mL, Medium BA); however, these differences were not found to respond in a dose-dependent manner to differences in BA content of the three protonated ePen3 samples. We therefore conclude that the presence of BA did not influence carbonyl emissions in this study. Taken as a whole, these data demonstrate the thermal stability of BA in a closed system e-cigarette, consistent with findings from a previous study (Pankow et al., 2017).

A concern regarding the use of acidic compounds in e-liquids is the potential for increased metal content of the resulting e-cigarette aerosol. However, our data from the accelerated aging test demonstrated that none of the metals, other than zinc, showed evidence for an impact of benzoic acid on metals emissions. In particular, it is notable that there was no observable increase in emissions of the coil metals Ni or Fe with increasing acid content. Naturally, if background levels could be reduced beyond those currently achievable then it may be possible to discern lower levels of metals potentially emitted by the e-cigarettes. Levels of metals in the e-liquids were not measured in this study, and it is possible that their metal ion concentrations may have changed in the aging tests, due to acid-mediated corrosion. However, if so, these metal ions did not (other than zinc) show increased transfer to the aerosol. The one metal showing an increased presence in the e-cigarette aerosols, zinc, is quoted as having a boiling point of 249°C in its dibenzoate form (CHEMSRC, 2020). Therefore, it is plausible that zinc dibenzoate could volatilise at e-cigarette operating temperatures. However, this reported boiling point value is not necessarily credible, as benzoic acid itself has a reported boiling point of 249°C (Alberty et al., 2007), and the quoted value for nickel benzoate is also 249°C (Guidechem, 2020). Nevertheless, the presence in the aerosol does indicate some degree of volatility at e-cigarette operating temperatures.

Sources of metals in e-cigarettes vapor and their potential health consequences were discussed by Williams et al. (2017). The presence of zinc was attributed to brass wire-to-wire clamp joints in atomisers within the e-cigarettes. Farsalinos et al. (2015), Williams et al. (2017), Olmedo et al. (2018), and Farsalinos et al. (2018) considered the hazards and risks associated with metal inhalation from e-cigarettes. Williams et al. (2017) and Olmedo et al. (2018) noted from established toxicological properties that inhalation of zinc from e-cigarettes carried potential hazards of metal fume fever, decreasing pulmonary function chest pain, coughing, dyspnea, and shortness of breath (ATSDR, 2005). However, Olmedo et al. (2018) noted that the established health effects for inhalation of zinc have arisen mostly in occupational settings during both acute and chronic exposures at relatively high levels. They concluded that these effects might not be relevant to chronic zinc exposure from e-cigarette use. Support for this view was provided by Farsalinos et al. (2015, 2018), who conducted risk assessments of daily zinc exposure from vaping and estimated that it was 6,000 times lower than the National Institute of Occupational Safety and Health (NIOSH)-established Relative Exposure Limit (REL). Using the data from the present study, without any background subtraction, would point to exposure at least 3,000 times lower than the REL. It is also notable that the zinc emissions per puff were four times lower from the highest BA containing e-cigarette than from cigarette smoke. Consequently, it appears that the zinc emissions measured in this study might not pose a significant risk to users of these e-cigarettes.

### Potential Contribution of a Cotton Wick and NiFe Coil to Non-metallic Toxicant Yields

Differences in aerosol chemistry between ePen3 (18 mg/mL, BT) and ePen2 (18 mg/mL, BT) provide a comparative examination of the contribution to toxicant emissions of, respectively, a cotton wick/NiFe coil e-cigarette design and a silica wick/NiCr coil design, although the comparisons are confounded to a degree by differences in the e-liquid composition (% PG/VG/water: ePen2 BT, 25/48/25; ePen3 BT, 54/34/10). As discussed in the introduction, cotton is hypothesized to be more thermally unstable than silica, resulting in higher emissions of carbonyls, acids and esters from low-temperature decomposition reactions (>180°C); higher levels of benzene, toluene, naphthalene (plus derivatives) and anthracene from mid-temperature reactions (>350°C); and greater PAH emissions from higher-temperature reactions (>400–500°C).

Comparison of potential low-temperature decomposition products between ePen3 BT and ePen2 BT did not support the hypothesis that emissions are higher in an e-cigarette with a cotton wick. Only isobutyraldehyde was significantly higher in emissions from the cotton wick/NiFe coil product. In contrast, formaldehyde, acetaldehyde, propionaldehyde, and acrolein were significantly higher in the aerosol from the silica wick/NiCr coil product, while the other carbonyls did not differ significantly between the two types of e-cigarette. The levels of acrolein, acetaldehyde, crotonaldehyde, formaldehyde, and propionaldehyde reported here for ePen3 are among the lowest reported in the literature, further supporting the use of cotton wick/NiFe coil as e-cigarette components that minimize toxicant yields (Belushkin et al., 2020; Münzel et al., 2020). The levels of esters and acetic acid did not differ between ePen3 BT and ePen2 BT, and emissions of propionic acid were lower from the cotton wick/NiFe coil product (ePen3 BT).

A similar conclusion was drawn from the mid- and high temperature potential decomposition products. Neither type of e-cigarette generated detectable levels of benzene, toluene, ethylbenzene or the smaller hydrocarbons 1,3-butadiene and isoprene. Furthermore, of the 23 PAHs examined, 18 showed no evidence of formation in e-cigarette aerosol, and none of the remaining five PAHs was significantly higher in aerosol from the cotton wick/NiFe coil product than in aerosol from the silica wick/NiCr coil product. Napthalene was the only PAH quantifiable in all samples, but there were no significant differences in emissions from any of the e-cigarettes.

Consequently, these measurements provide no evidence for thermal decomposition reactions of cotton in the ePen3 e-cigarette, with the implication that for well-designed and manufactured devices, cotton wicks are stable under standard e-cigarette operating conditions. The data also provide no evidence for a significant influence of the metallic NiFe coil on carbonyl emissions. Thermal decomposition products of PG and VG, such as propylene oxide, glycolaldehyde, glyoxal, and methyl glyoxal, were not higher in the emissions from ePen3 BT than in those from ePen2. Hence, we conclude that a cotton wick/NiFe coil is suitable for use in a low-toxicant-emission e-cigarette design. The cytotoxicity of ePen3 has been compared to a reference cigarette and an earlier generation of open-tank e-cigarette, with clear differences in cytotoxic profiles reported between the two e-cigarettes (Bishop et al., manuscript in preparation). Full toxicity was achieved with 120 puffs from the open-tank device whereas a full cytotoxic curve was not achieved for ePen3 using 1,000 puffs, further supporting the use of cotton wick/NiFe coil technology.

### Analysis of the Additional 19 HPHCs Proposed by the FDA

The 19 additional compounds that the FDA has proposed adding to established lists of HPHCs in tobacco comprise a number of flavor compounds, aerosol formers, thermal decomposition products and contaminants in e-liquid components (FDA, 2019).

Among the flavor compounds, propionic acid (acidic, sweet, nutty aroma) was quantifiable in the emissions from ePen2 BT at 155 ng/puff, detected but not quantified in ePen3 BT, and not observed in the other three e-cigarette aerosols. The source of this compound is unclear because propionic acid is not a component of the Blended Tobacco flavor; however, its presence in the aerosol of both of the Blended Tobacco but none of the Master Blend e-cigarettes suggests that it is a flavor-related source. Only one other study has assessed propionic acid emissions from an e-cigarette, reporting values of 1.95–9.01 ng/puff (depending on puffing flowrate) from a refillable tank style e-cigarette (Kim and Kim, 2015). Those values are below the LOD of the method used in the current study (36 ng/puff). The present study laboratory did not have an established method for measuring propionic acid in cigarette smoke; however, published smoke data, ranging from 118 to 235 μg/cigarette (~10–25 μg/puff) (Buyske et al., 1957) to 300 μg/cigarette (Quin et al., 1961), are substantially higher than the value of 155 ng/puff measured in ePen2 BT aerosol (equating to a ~98–99% reduction).

The flavor compound ethyl acetate (ethereal, fruity, brandy-like aroma) was detected in the air/method blank and each of the e-cigarette aerosols at levels <LOQ. Thus, the presence of this compound seems to arise from contamination sources. To our knowledge, no other studies have reported ethyl acetate emissions from e-cigarettes, although one study identified (but did not quantify) ethyl acetate in aerosol samples (Uchiyama et al., 2016). However, ethyl acetate has been identified in e-liquids (Lim and Shin, 2013; Varlet et al., 2015; Tierney et al., 2016; Behar et al., 2018; LeBouf et al., 2018; My et al., 2019; Omaiye et al., 2019) and therefore is likely to be present in aerosols from some e-cigarettes.

Acetic acid and the remaining acetates on the additional FDA list (methyl acetate, ethereal fruity aroma; isobutyl acetate, fruity aroma; isoamyl acetate, banana/pear aroma; benzyl acetate, berry, sweet aroma; and ethyl acetoacetate, fruity aroma) were not detected in any of the e-cigarette aerosols or the air/method blanks. Similarly, none of the other flavourants (1-butanol, potato-like aroma; furfural, almond, bread, burnt, spice aroma) and flavor compounds (acetoin, butter aroma; acetyl propionyl, buttery, caramel, creamy aroma) were detected in any of the samples. Diacetyl (butter, butterscotch aroma) was not detected in the four ePen3 samples, but was detected at <LOQ (<5.8 ng/puff) in the ePen2 sample. It is not a component of e-liquids, so the reason for its presence in the ePen2 aerosol is unclear. Levels of acetoin, acetyl propionyl and diacetyl in the e-cigarette aerosols were reduced by >99.9% as compared with the cigarette smoke of both cigarettes. The complex chemistry of these three compounds in e-liquids has recently been investigated (Vas et al., 2019).

Among the aerosol formers and thermal decomposition products proposed by the FDA (FDA, 2019), VG, and PG were identified in all e-cigarette emissions. They are the main components of e-liquids and were present in substantially greater amounts in the aerosols than in cigarette smoke. Glycidol, the thermal decomposition product of VG, was not detected in the air/method blank, the e-cigarette aerosols, or 1R6F smoke; however, it was detected and quantified in B&H Skyblue cigarette smoke.

Lastly, diethylene glycol and ethylene glycol are hazardous compounds that have been found in e-liquids either as replacements of or contaminants in VG or PG. In this study, diethylene glycol was not detected in any e-cigarette sample, while ethylene glycol was detected in two of the five aerosol samples at an average of 0.0045% of the level of PG and VG emissions. Thus, use of pharmaceutical grade PG and VG in these e-cigarettes seems to minimize contamination by diethylene glycol and ethylene glycol.

The above findings suggest that, other than VG and PG, the additional 19 HPHC compounds proposed for inclusion on the FDA's established list of HPHCs are not common in e-cigarette emissions. Apart from PG and VG, only one of the compounds, propionic acid, was quantified in the e-cigarette aerosols in the present study. The majority of the proposed 19 HPHCs are flavourants, most of which provide fruity or buttery flavors; therefore, they may be more likely to be found only in specific kinds of flavored e-liquid. There is no evidence that they are thermally generated, and thus the likelihood of their presence in e-cigarette aerosols is likely to be dictated by whether they are chosen by manufacturers as flavor ingredients in the e-liquids. Studies of diacetyl, acetyl propionyl and acetoin in e-liquids have shown that such ingredients can transfer efficiently to the aerosol (Farsalinos et al., 2015), and can in some circumstances arise from the use of other ingredients (Vas et al., 2019). The present findings also suggest that the presence of glycol contaminants can be minimized or avoided by using pharmaceutical purity PG and VG, in-line with EU purity standards (EU, 2014).

### Comparison to Cigarettes

In almost every case, per-puff cigarette yields of the 84 toxicants examined for both types of product were substantially higher than per-puff aerosol yields from the e-cigarettes. The same behavior was observed when emissions were compared on a per-nicotine basis. Two clear exceptions were PG and VG, which were higher in e-cigarette emissions than in cigarette smoke. This is because PG and VG are the major e-liquid and aerosol components used in these products, comprising 85–90% of both matrices. These compounds are not classified in terms of toxicity and their inhalation toxicology has been studied without identification of significant concerns for users (Cotta et al., 2017; Phillips et al., 2017), however their long-term use warrants further investigation. One of the e-cigarettes (ePen3 30 mg/mL, High BA) also gave higher nicotine emissions per puff than from the cigarettes The impurity ethylene glycol was quantified in two e-cigarettes but not detected in smoke from the cigarettes.

Comparing the impact of comparisons made per-puff to those made per-nicotine showed relatively little impact across all of the toxicants examined in this study. This is because of the significant number whose emissions were too low to quantify or were not detectable in the e-cigarette aerosols.

However, focusing solely on those toxicants which were quantifiable did show some differences between the comparison methods. Nicotine emissions from the study products ran in the following order (values in backets are the rounded nicotine emissions per puff in μg): ePen2 18BT (40) < ePen3 12 low BA (131) < ePen3 18 BT (149) < ePen3 18 Medium BA (168) < 1R6F (210) = B&H Skyblue (210) < ePen3 MB 30 high BA (256). Therefore, nicotine emissions varied more than 6-fold amongst this sample set, with a mean value (166) very close to that of ePen3 18 Medium BA (168). Hence, relative to this mid-point product, calculating toxicant emission values per nicotine raised the values from epen2 product and both the ePen3 12 mg low BA and 18 mg (no BA) nicotine products, while reducing the values from the highest nicotine content ePen3 product and the two cigarettes. The impact of this calculational approach was most significant with the ePen2 product. Consequently, with the quantifiable toxicant/nicotine emissions reported in Table 4, ePen2 values are greater (whether significantly or not) than all of the quantified compounds other than PG. Under the per-nicotine model ePen2 would therefore provide greater estimated toxicant exposure than the epen3 products despite the greater mass of aerosol generated by the ePen3 products.

In contrast, comparing the toxicant/nicotine values in Table 4 from the e-cigarettes to cigarette smoke, showed that apart from water, ACM/NFDPM, PG, and VG, all of the other analytes were lower from every tested e-cigarette than from the combustible tobacco cigarettes (including the many toxicants whose emissions were too low to quantify or detect in the e-cigarette aerosols). Therefore, use of either per-puff or per-nicotine calculations points to lower levels of toxicant emissions from these e-cigarettes than from cigarette smoke.

In the present study, we quantified the relative difference in toxicants between e-cigarette emissions and cigarette smoke by calculating percentage reductions. Such calculations are challenged by the fact that many e-cigarette emissions are too low to quantify. A number of approaches have been adopted to impute non-quantifiable values in different datasets, including the midpoint approach used in the present study (Margham et al., 2016), use of LOD/√2, predicted values from models, and use of sub-detection limit values presented by the analytical method (Succop et al., 2004).

To assess the effect or potential errors brought about by use of the midpoint imputation approach taken in this study, we re-calculated the percent reductions for the TobReg 9 priority toxicants (Burns et al., 2008) using two boundary conditions—upper and lower boundary values (Table 6). Regardless of whether 1R6F or B&H Skyblue was used as the reference cigarette smoke sample, highly similar percent reductions were obtained by all three approaches. The differences between the upper boundary and lower boundary approaches were <0.2% (e.g., 99.89% average reduction with the lower boundary estimate and 99.73% with the upper boundary estimate). Because all unquantifiable values must lie between these extremes, it is clear that the reductions in the WHO TobReg 9 toxicant emissions between cigarette smoke and e-cigarette aerosol are so substantial that imputation errors are trivial. Given these findings, we regard the midpoint imputation approach as an appropriate strategy. Use of this strategy shows that, on average, emissions of the WHO TobReg 9 analytes are >99% lower from all tested e-cigarettes, whether compared with the reference product 1R6F or the commercial cigarette B&H Skyblue (Table 6).

**Table 6:** Per-puff % reductions in WHO TobReg 9 constituents from ePen e-cigarettes relative to combustible cigarette emissions estimated by the mid-point estimation approach and two boundary conditions for unquantifiable and undetectable toxicants.

**% Reductions in comparison to 1R6F**															
**Toxicant**	**ePen2 BT 18**			**ePen3 BT 18**			**ePen3 MB 12 Low BA**			**ePen3 MB 18 Medium BA**			**ePen3 MB 30 High BA**		
	Lower^*^	**Mid** ^**†**^	**Upper** ^**‡**^	Lower^*^	**Mid** ^**†**^	**Upper** ^**‡**^	Lower^*^	**Mid** ^**†**^	**Upper** ^**‡**^	Lower^*^	**Mid** ^**†**^	**Upper** ^**‡**^	Lower^*^	**Mid** ^**†**^	**Upper** ^**‡**^
CO	>99.9	99.82	99.64	>99.9	99.82	99.64	>99.9	99.82	99.64	>99.9	99.82	99.64	>99.9	99.82	99.64
Acetaldehyde	99.86	99.86	99.86	99.99	99.99	99.98	99.94	99.94	99.94	99.99	99.99	99.98	99.98	99.98	99.98
Acrolein	97.61	97.61	97.61	100	99.97	99.94	>99.9	99.97	99.94	99.94	99.86	99.79	99.94	99.86	99.79
Formaldehyde	94.51	94.51	94.51	98.92	98.92	98.92	96.33	96.33	96.33	97.76	97.76	97.76	97.47	97.47	97.47
Benzo[a]pyrene	>99.9	99.69	99.38	>99.9	99.69	99.38	>99.9	99.69	99.38	>99.9	99.69	99.38	>99.9	99.69	99.38
NNK	>99.9	99.96	99.93	>99.9	99.96	99.93	>99.9	99.96	99.93	>99.9	99.96	99.93	>99.9	99.96	99.93
NNN	>99.9	99.98	99.96	>99.9	99.98	99.96	>99.9	99.98	99.96	>99.9	99.98	99.96	>99.9	99.98	99.96
Benzene	>99.9	99.98	99.96	>99.9	99.98	99.96	>99.9	99.98	99.96	>99.9	99.98	99.96	>99.9	99.98	99.96
1,3-Butadiene	>99.9	99.97	99.94	>99.9	99.97	99.94	>99.9	99.97	99.94	>99.9	99.97	99.94	>99.9	99.97	99.94
Mean estimate	99.11	99.04	98.98	99.88	99.81	99.74	99.59	99.52	99.45	99.74	99.67	99.59	99.71	99.64	99.56
**% Reductions in comparison to B&H Skyblue**															
**Toxicant**	**ePen2 BT 18**			**ePen3 BT 18**			**ePen3 MB 12 Low BA**			**ePen3 MB 18 Medium BA**			**ePen3 MB 30 High BA**		
	Lower^*^	**Mid** ^**†**^	**Upper** ^**‡**^	Lower^*^	**Mid** ^**†**^	**Upper** ^**‡**^	Lower^*^	**Mid** ^**†**^	**Upper**	Lower^*^	**Mid** ^**†**^	**Upper** ^**‡**^	Lower^*^	**Mid** ^**†**^	**Upper** ^**‡**^
CO	>99.9	99.81	99.62	>99.9	99.81	99.62	>99.9	99.81	99.62	>99.9	99.81	99.62	>99.9	99.81	99.62
Acetaldehyde	99.87	99.87	99.87	99.99	99.99	99.98	99.94	99.94	99.94	99.99	99.99	99.98	99.98	99.98	99.98
Acrolein	97.83	97.83	97.83	100	99.97	99.94	100	99.97	99.94	99.94	99.87	99.81	99.94	99.87	99.81
Formaldehyde	94.88	94.88	94.88	98.99	98.99	98.99	96.58	96.58	96.58	97.91	97.91	97.91	97.64	97.64	97.64
Benzo[a]pyrene	>99.9	99.71	99.43	>99.9	99.71	99.43	>99.9	99.71	99.43	>99.9	99.71	99.43	>99.9	99.71	99.43
NNK	>99.9	99.92	99.83	>99.9	99.92	99.83	>99.9	99.92	99.83	>99.9	99.92	99.83	>99.9	99.92	99.83
NNN	>99.9	99.95	99.89	>99.9	99.95	99.89	>99.9	99.95	99.89	>99.9	99.95	99.89	>99.9	99.95	99.89
Benzene	>99.9	99.98	99.96	>99.9	99.98	99.96	>99.9	99.98	99.96	>99.9	99.98	99.96	>99.9	99.98	99.96
1,3-Butadiene	>99.9	99.97	99.94	>99.9	99.97	99.94	>99.9	99.97	99.94	>99.9	99.97	99.94	>99.9	99.97	99.94
Mean estimate	99.18	99.1	99.03	99.89	99.81	99.73	99.61	99.54	99.46	99.76	99.68	99.6	99.73	99.65	99.57

*BT, Blended Tobacco; MB, MasterBlend, BA, benzoic acid*.

* *Lower boundary estimate approach, where results <LOD are taken as 0 and <LOQ values are taken as LOD*.

*Mid-point estimate, where results <LOD are estimated as LOD/2, and results <LOQ are estimated as (LOD+LOQ)/2*.

‡ *Upper boundary estimate approach, where <LOD = LOD and <LOQ = LOQ*.

Other calculational approaches to compare emissions, such as subtraction of air/method blank levels, use of per-day rather than per-puff exposure estimates, and per-nicotine values might be considered. However, the subtraction approach potentially compounds errors in cases where the air/method blank values are <LOQ and <LOD and need to be subtracted from e-cigarette values that are also <LOD or <LOQ. Use of per-day estimates are also prone to errors due to uncertainties in consumption values for cigarettes and e-cigarettes. As noted above, similar estimates exist for e-cigarette and combustible cigarettes puffs per day, therefore per-day reductions values might be similar to per-puff reductions. However, there are significant uncertainties in the values obtained by using these calculations. Finally, calculating the % reductions using per-nicotine rather than per-puff data leads to very similar conclusions. With ePen2 18 BT the % reductions (against 1R6F, B&H Skyblue) per nicotine are (94.9, 95.3%) compared to (99, 99.1%) per puff; with ePen3 BT 18 per nicotine (99.7, 99.7%), per puff (99.8, 99.8%); with ePen3 MB 12 Low BA per nicotine (99.2, 99.3%) and per puff (99.5, 99.5%); with ePen3 MB 18 Medium BA per nicotine (99.6, 99.6%) and per puff (99.7, 99.7%); with ePen3 MB 30 High BA per nicotine (99.7, 99.7%) and per puff (99.6, 99.7%). The very similar values arise because most of the TobReg9 analytes are either <LOD or <LOQ with the e-cigarettes, and therefore so low in comparison to cigarette smoke values that they show little sensitivity to normalization by puff or by nicotine.

### Study Limitations

The experimental design for comparison of emissions between products with differing wick designs was not ideal through practical necessity. Due to significantly differing wicking/viscosity properties the same e-liquid composition could not be used with the two materials. We matched compositions as closely as possible and used e-liquids that would typically be encountered with commercial examples of both wicking systems, but nevertheless comparisons of aerosol emissions were not straightforward. There were also device design and power setting differences between the products used. However, the aerosols from these devices showed an absence of marker compounds for thermal degradation of cotton, metal-catalyzed PG/VG degradation, or acid mediated coil corrosion. Moreover, despite the higher power and aerosol/puff of the cotton wicked device emissions per puff were not elevated in comparison to the silica wicked product. These findings clearly demonstrate that e-cigarette designs can be developed with cotton wicks, NiFe coils and nicotine benzoate without compromising the low levels of toxicant emissions that can be achieved with e-cigarettes.

Although the toxicant emissions from the e-cigarettes showed substantial reductions in comparison to combustible tobacco cigarettes, a study focusing on aerosol chemistry cannot fully investigate the health risks associated with e-cigarette use. Consideration also needs to be given to potential health effects of long-term exposure to the major aerosol components (Stratton et al., 2018), potential aging effects with open device e-cigarette performance over time, the effect on toxicant exposure arising from natural user variation in vaping behaviors (McAdam et al., 2019) and consumption levels. Also, concerns over the consequences of nicotine exposure arising from use of nicotine salts require further investigation (CNBC, 2019; CDC, 2020).

## Conclusions

We have conducted a comparative study analyzing toxicant emissions from five e-cigarettes and two tobacco cigarettes, wherein 97 aerosol constituents and 84 smoke compounds, respectively, were quantified. The data obtained have enabled us to examine several emerging issues in e-cigarette science, namely whether the introduction of recent product features such as cotton wicks, NiFe coils and nicotine benzoate produce differences in aerosol chemistry in comparison to older design alternatives. Targeted analyses of marker compounds for thermal degradation of cotton wicks and nicotine benzoate showed no evidence for their breakdown during e-cigarette use. Similarly, use of a NiFe coil neither lead to enhanced decomposition of the major aerosol constituents, nor increased metal content of the aerosol (other than small increases in zinc) despite concerns of acid-mediated coil corrosion. Comparison to cigarette smoke emissions demonstrated that e-cigarettes containing these recent design features can offer 99% reductions in priority smoke toxicants. Finally, the absence of any of the FDA proposed 19 additional HPHCs (other than PG, VG and propionic acid) from these e-cigarettes suggest that the presence of these compounds in e-cigarette aerosols will be largely dictated by manufacturers ingredient choices.

## Data Availability Statement

The original contributions presented in the study are included in the article/supplementary materials, further inquiries can be directed to the corresponding author/s.

## Author Contributions

AC managed the analytical chemistry program and co-authored the article. KM wrote the manuscript. JT conducted the data analysis. HD directed the study and was responsible for product integrity. All authors contributed to the article and approved the submitted version.

## Conflict of Interest

British American Tobacco (BAT) was the funding organization for the study. During the course of the study and preparation of the manuscript BAT paid McAdam Scientific Ltd. for the consultancy services of KM, who is also the owner of McAdam Scientific Ltd.; the remaining authors were paid employees of BAT.
